# Nanotechnology-driven strategies for gene therapy of arthritis

**DOI:** 10.1016/j.mtbio.2025.102301

**Published:** 2025-09-11

**Authors:** Shuo Wang, Yuequan Wang, Qin Chen

**Affiliations:** a Department of Pharmacy, Cancer Hospital of China Medical University, Liaoning Cancer Hospital & Institute, No. 44 Xiaoheyan Road, Dadong District, Shenyang, 110042, Liaoning Province, China; b Department of Pharmaceutics, Wuya College of Innovation, Shenyang Pharmaceutical University, Shenyang, Liaoning, 110016, China

**Keywords:** Gene therapy, Nanotechnology, Arthritis, Targeted drug delivery, CRISPR-Cas9

## Abstract

Arthritis remains a major global health burden, driven by chronic inflammation and irreversible joint damage, with current treatments often failing to achieve sustained remission. Gene therapy offers a promising strategy to address the root molecular causes by reprogramming dysregulated immune responses and promoting tissue regeneration. However, clinical translation is limited by delivery inefficiency, off-target effects, and immunogenicity. Recent advances in genetic technologies, including RNA-based therapies, DNA-based strategies, and CRISPR-Cas9 gene editing, have broadened the therapeutic toolkit for precise and durable gene modulation. Concurrently, the integration of nanotechnology has enabled the development of smart delivery platforms, such as lipid nanoparticles, polymeric carriers, inorganic nanostructures, extracellular vesicles, membrane-coated systems, and DNA nanoframeworks, to overcome biological barriers and enhance gene transfection. Moreover, emerging therapeutic strategies targeting immune modulation, synovial macrophage reprogramming, inflammatory signaling, cartilage preservation and regeneration, and pain relief further expand the clinical potential. This review provides a comprehensive overview of the key genetic tools, nanoplatforms, and therapeutic approaches driving next-generation gene therapy for arthritis, highlighting a multidisciplinary path toward precise, effective, and long-lasting treatment.

## Highlights

• Comprehensive overview of cutting-edge genetic technologies for precise arthritis gene modulation.
• Detailed analysis of nanotechnology-based delivery platforms overcoming biological barriers for targeted gene therapy.
• Exploration of novel therapeutic strategies focusing on immune regulation, cartilage protection, and pain relief.
• Insightful discussion on current challenges and future directions for clinical translation.

## Graphical abstract

**Figure:**
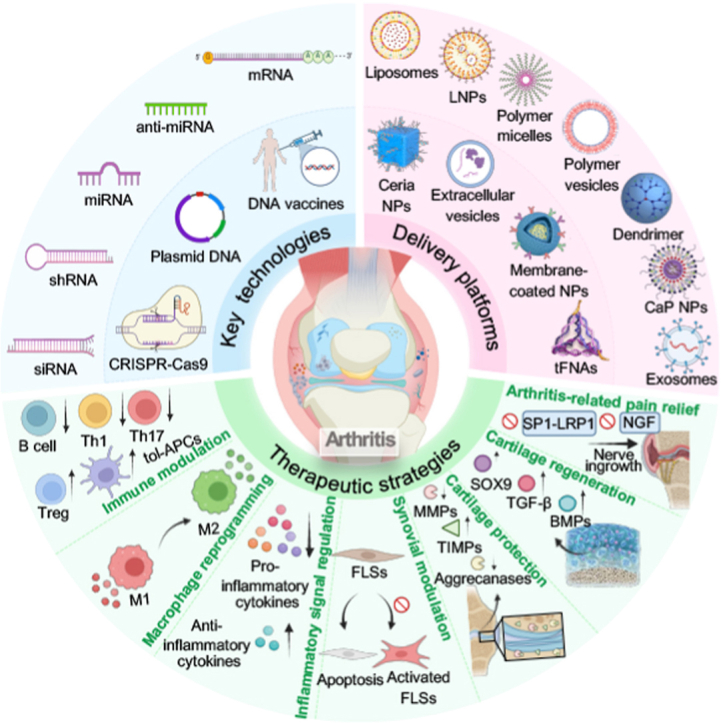

## 1 Introduction

Arthritis, a group of chronic inflammatory joint disorders, represents a significant global health burden, affecting over 350 million individuals worldwide and contributing substantially to disability and reduced quality of life [[1], [2], [3], [4]]. The two most prevalent forms, rheumatoid arthritis (RA) and osteoarthritis (OA), are characterized by distinct yet overlapping pathological mechanisms. RA is an autoimmune disease (AID) driven by dysregulated adaptive immune responses, with synovial hyperplasia and pannus formation invading cartilage and bone, ultimately leading to severe joint erosion and systemic complications [5,6]. In contrast, OA is primarily a degenerative condition associated with progressive cartilage breakdown, subchondral bone remodeling, osteophyte formation, and low-grade, innate immune–driven inflammation [7]. Despite these differences, RA and OA share common pathological features, including synovial inflammation, the release of pro-inflammatory cytokines such as TNF-α, IL-1β, and IL-6, and cartilage matrix degradation mediated by matrix metalloproteinases (MMPs). Clinically, both result in chronic pain, joint dysfunction, and irreversible structural damage, posing significant challenges for long-term management. Current therapies, such as nonsteroidal anti-inflammatory drugs (NSAIDs), disease-modifying antirheumatic drugs (DMARDs), and biologic agents (e.g., TNF-α inhibitors), primarily focus on symptom management rather than curative outcomes [8,9]. However, these approaches often suffer from limited efficacy, systemic side effects, and high costs [10], underscoring the urgent need for innovative therapeutic strategies that can provide long-term solutions.

In recent years, gene therapy has emerged as a promising frontier in the treatment of arthritis, offering the potential to target the underlying molecular mechanisms of disease. By delivering therapeutic genes to specific cells or tissues, gene therapy aims to modulate inflammatory pathways, promote tissue repair, and restore joint homeostasis. For instance, the use of viral vectors, such as adeno-associated viruses (AAVs), to deliver anti-inflammatory cytokines (e.g., IL-10 or TGF-β) has shown encouraging results in preclinical models of RA [11]. Similarly, CRISPR-Cas9-based gene editing has been explored to silence pro-inflammatory genes (e.g., TNF-α or IL-6) or correct genetic mutations associated with arthritis [12]. Despite these advances, the clinical translation of gene therapy faces significant challenges, including inefficient delivery to target tissues, immune responses to viral vectors, and potential off-target effects [13,14]. These limitations highlight the need for innovative delivery systems that can enhance the precision, safety, and efficacy of gene-based interventions.

The integration of nanotechnology with gene therapy has opened new avenues for overcoming these challenges, offering unprecedented opportunities for targeted and controlled delivery of therapeutic genes. Nanotechnology leverages the unique physicochemical properties of nanoscale materials to design advanced delivery systems capable of navigating biological barriers and achieving site-specific gene expression [[15], [16], [17], [18], [19]]. For example, lipid nanoparticles (LNPs) and polymeric nanoparticles (PNPs) have been extensively studied for their ability to encapsulate and protect nucleic acids, such as small interfering RNA (siRNA), messenger RNA (mRNA), or CRISPR components, while facilitating their uptake by target cells [[20], [21], [22]]. LNPs, in particular, have gained significant attention due to their success in mRNA-based COVID-19 vaccines, demonstrating their potential for clinical translation [23]. Additionally, surface modification of nanoparticles (NPs) with targeting ligands (e.g., peptides, antibodies, or aptamers) enables precise delivery to inflamed joints or cartilage, minimizing off-target effects [24,25]. Recent advances in stimuli-responsive nanomaterials, which release their payload in response to specific triggers (e.g., pH, redox potential, or enzymes), further enhance the spatiotemporal control of gene delivery, ensuring therapeutic effects are confined to diseased tissues [26]. Moreover, nanotechnology offers unique advantages in addressing the complex pathophysiology of arthritis. For instance, the dense extracellular matrix (ECM) of cartilage poses a significant barrier to the penetration of therapeutic agents [27]. NPs, due to their small size and tunable surface properties, can overcome this barrier and deliver genes directly to chondrocytes or synovial cells [28]. Furthermore, nanotechnology enables the co-delivery of multiple therapeutic agents, such as genes and small-molecule drugs, to achieve synergistic effects. For example, a single nanocarrier could deliver siRNA to silence a pro-inflammatory gene while simultaneously releasing an anti-inflammatory drug to suppress local inflammation [29]. This combinatorial approach not only enhances therapeutic efficacy but also reduces the risk of drug resistance and systemic toxicity.

**Fig. 1:**
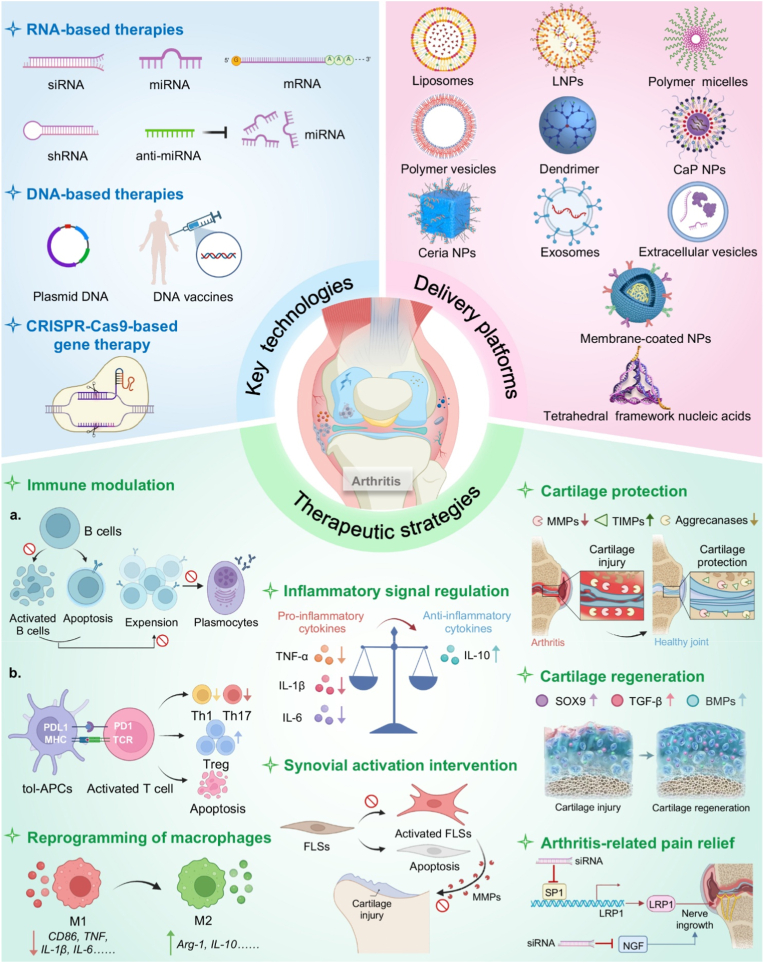
Schematic illustration of gene therapy for arthritis: key technologies, delivery platforms, and therapeutic strategies.

The convergence of gene therapy and nanotechnology holds immense promise for revolutionizing the treatment of arthritis [30,31]. By combining the precision of gene-based interventions with the versatility of nanoscale delivery systems, this interdisciplinary approach addresses critical limitations of conventional therapies and paves the way for personalized and regenerative medicine. For instance, nanotechnology-enabled delivery of CRISPR-Cas9 components can achieve targeted gene editing in fibroblast-like synoviocytes (FLSs) or chondrocytes, offering the potential for long-term remission or even cure [32,33]. Similarly, the co-delivery of genes and anti-inflammatory drugs using multifunctional nanocarriers can synergistically suppress inflammation and promote tissue repair [29]. As research in this field continues to advance, the development of biocompatible, scalable, and clinically translatable nanoplatforms will be essential for realizing the full potential of this transformative approach. This review systematically explores the cutting-edge developments in arthritis gene therapy, with a particular focus on three key aspects: (1) the foundational genetic technologies enabling therapeutic intervention, (2) innovative nanoplatforms for targeted gene delivery, and (3) emerging therapeutic strategies for disease modification. We begin by examining the latest RNA-based, DNA-based, and CRISPR-Cas9 gene editing technologies that form the basis of modern arthritis gene therapy. The discussion then shifts to advanced delivery systems, including lipid-based NPs, polymeric carriers, inorganic nanomaterials, and novel biomimetic platforms, analyzing their respective advantages and challenges in joint-targeted delivery. Finally, we comprehensively review therapeutic approaches targeting various pathological aspects of arthritis, from immune modulation and macrophage reprogramming to cartilage protection and regeneration (Fig. 1). By integrating these multidisciplinary advances, this review aims to provide researchers and clinicians with a comprehensive understanding of how gene therapy is transforming arthritis treatment, while also highlighting remaining challenges and future directions in this rapidly evolving field.

## 2 Key genetic technologies enabling gene therapy

The remarkable progress in gene therapy for arthritis has been driven by revolutionary advances in genetic engineering technologies, which can be broadly categorized into RNA-based, DNA-based, and CRISPR-Cas9-based approaches. These technologies enable precise modulation of disease-related genes, offering unprecedented opportunities to target the underlying molecular mechanisms of arthritis rather than merely alleviating symptoms. RNA-based strategies, including siRNA, short hairpin RNA (shRNA), and microRNAs (miRNAs), which enable selective silencing of pro-inflammatory mediators, while mRNA serves as a platform to supplement therapeutic protein expression [29,[34], [35], [36]]. DNA-based therapies utilize viral and non-viral vectors to deliver genes aimed at sustained therapeutic effects [37]. Most notably, the emergence of CRISPR-Cas9 gene editing has opened new possibilities for correcting disease-causing mutations or epigenetically reprogramming pathological cellular processes [38]. Each of these technologies possesses unique advantages and challenges in terms of specificity, durability, and translational potential. The sections will systematically discuss their mechanisms, applications, and recent advancements in the context of arthritis therapy, providing a foundation for understanding how genetic interventions can be harnessed to combat joint degeneration and inflammation (Table 1).

**Table 1:** Key genetic technologies enabling gene therapy.

**Genetic technology**	Formulations	Molecular mechanism	Models	Refs
RNA-based gene therapy	M2H@RPK	Leptin signaling blockade (shRNA-LEPR)	OA (ACLT + DMM surgery)	[29]
	Exos^*shEZH*2^@HG	Silencing EZH2 (shRNA-EZH2)	CIA	[39]
	shTACE/(8D-16R) complex	Against TNF-α converting enzyme (shRNA-TACE)	CIA	[40]
	MTX + RELA siRNA FOL-Liposome	Silencing NF-κB (siRNA-RELA)	CIA	[41]
	mAbCII-siNPs	Silencing MMP13 (siRNA-MMP13)	PTOA	[34]
	MACP siTNF-α NPs	Silencing TNF-α gene (siRNA-TNF-α)	CIA	[42]
	ICPm21	Silencing PDCD4 and inhibiting NF-κB (miRNA-21)	ZIA	[43]
	miR-17-5p lipoplex	Disrupting the IL-6/JAK/STAT autocrine loop (miRNA-17-5p)	CIA	[44]
	M-PHMs/miR-124	Silencing NFATc1 and blocking osteoclast (miRNA-124)	AIA	[45]
	G5-AHP/miR-224-5p NPs	Targeting PTX3 (miRNA-224-5p).	OA (DMM surgery)	[46]
	T-miR3 (tFNAs-miR-124)	Suppressing TNF-α/IL-6/MMPs and upregulating COL2/AGC/SOX9 (miRNA-124)	OA (ACLT + DMM surgery)	[47]
	CAP-PVAm-PLGA NPs	Silencing ADAMTS-5/MMP13 and upregulating COL2A1 (miRNA-140)	OA (DMM surgery)	[48]
	PEG-Liposomal AntagomiR-155-5p	Reversing defective M2 polarization via SOCS-1/CEBPβ (anti-miRNA-155-5p)	CIA + STA	[49]
	CS-CDs@155	Promoting M1-to-M2 polarization (anti-miRNA-155)	AIA	[50]
	YCWP-NPs@miR365 antagomir	Reducing inflammation and cartilage degradation (anti-miRNA-365)	PTOA	[51]
	LNPs/mPDL1	Inducing PDL1 expression to suppress overactivated T cells via the PDL1/PD1 pathway (mRNA-PDL1)	CIA	[52]
	MR@P-mPTEN	Restoring PTEN expression and inhibiting PI3K/AKT pathway activation (mRNA-PTEN)	CIA	[53]
DNA-Based Therapies	bPEI-SS-PEG-T/NLS/DNA NPs	Promoting M1-to-M2 polarization via mTOR pathway inhibition and Arg2 induction (pDNA-IL-10)	CIA	[54]
	pPNP + TIIA@PFS hydrogel	Expressing Klotho (anti-aging protein; Klotho-pDNA)	OA (ACLT surgery)	[55]
	M2 Exo/pDNA/BSP NPs	Promoting M1-to-M2 polarization increasing the expression of IL-10 cytokine (pDNA-IL-10)	CIA	[56]
	pcDNA-CCOL2A1 vaccine	Transiently expressing CCII mRNA and protein (pcDNA-CCOL2A1; DNA vaccine)	–	[57]
CRISPR-Cas9-based gene therapy	SMART-Cas9	Knocking out RhoA, disrupting cytoskeletal dynamics and osteoclast differentiation (RhoA-targeting CRISPR-Cas9 plasmid)	CIA	[33]
	hybrid CAP-Exo	Knocked down MMP13 (MMP13-targeting CRISPR-Cas9 plasmid)	OA	[58]
	NP_Cas9/gB220_	Disrupting B220 expression in B cells (pX330 plasmid (Cas9 + gB220 gRNA))	CIA	[59]
	CAP/FGF18-hyEXO@HMs	Enabling precise genome-level FGF18 activation in OA chondrocytes (Cas9-sgFGF18 plasmid (pX330 backbone))	OA	[60]

### 2.1 RNA-based gene therapy

RNA-based therapies have emerged as a powerful and versatile platform in gene therapy for arthritis, offering the ability to modulate gene expression at the post-transcriptional level with high specificity. Unlike traditional pharmacological interventions that often act indirectly and non-selectively, RNA therapeutics directly target the molecular drivers of disease, providing opportunities for precision intervention in complex inflammatory and degenerative pathways [61]. In the context of arthritis, RNA molecules can serve distinct therapeutic roles depending on their structure and mode of action. siRNAs and shRNAs are widely utilized for gene silencing, enabling the targeted knockdown of pro-inflammatory or cartilage-degrading genes [29,34]. miRNAs, as endogenous regulators, exert broader effects by modulating networks of genes involved in inflammation, tissue remodeling, and pain signaling [35]. Meanwhile, mRNAs represent a strategy for restoring or enhancing the expression of protective or reparative proteins, particularly in conditions where beneficial gene function is compromised [36]. Together, these RNA-based approaches provide a comprehensive toolkit to either suppress pathogenic mechanisms or augment regenerative pathways, laying the foundation for next-generation gene therapies in arthritis.

Among various RNA-based gene silencing tools, siRNA offers high specificity in downregulating disease-driving genes through the RNA interference (RNAi) pathway. By guiding the RNA-induced silencing complex (RISC) to complementary mRNA targets, siRNA induces mRNA degradation, effectively suppressing the expression of pathogenic proteins [62]. In inflammatory arthritis, the persistent activation of transcription factors such as NF-κB drives the expression of multiple pro-inflammatory cytokines and enzymes, contributing to synovial hyperplasia, immune cell infiltration, and joint destruction [63]. Targeting upstream nodes in this cascade provides an opportunity for efficient inflammation resolution with minimal off-target effects. Notably, RELA, encoding the p65 subunit of NF-κB, serves as a central regulator in this axis [64]. Direct silencing of RELA via siRNA has the potential to shut down the entire pro-inflammatory transcriptional program. To harness this therapeutic potential, Nasra et al. developed a folate-targeted liposomal nanoformulation co-delivering methotrexate (MTX) and RELA siRNA for RA therapy [41]. The liposomes were synthesized using DSPC, cholesterol, and DSPE-PEG-folate, optimized via a Box-Behnken design, and characterized for size (≈140 nm), stability, and high encapsulation efficiency (*EE*%, 96 % for MTX, 93 % for siRNA) [41]. The folate ligand enabled targeted delivery to macrophages, while the RELA siRNA silenced NF-κB, synergizing with MTX to repolarize pro-inflammatory M1 macrophages to anti-inflammatory M2 phenotypes [41]. *In vitro* and *in vivo* studies demonstrated reduced synovial inflammation, decreased pro-inflammatory cytokines (TNF-α, IL-6), and improved joint mobility in collagen-induced arthritic (CIA) rats [41]. This approach highlights RNAi's precision in modulating inflammatory pathways, offering a potent, targeted strategy for RA with reduced systemic toxicity.

**Fig. 2:**
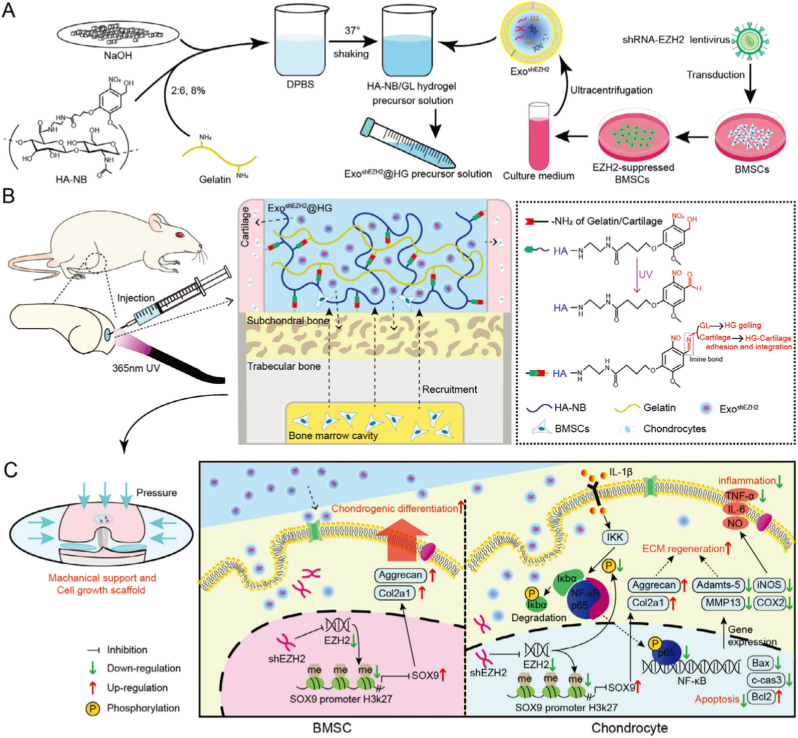
Overview of the synthesis and mechanism of action of Exo^shEZH2^@HG. Reproduced with permission from Ref. [39]. Copyright 2025 Wiley-VCH GmbH.

While siRNA provides rapid and transient gene silencing, shRNA offers a more sustained and stable approach to gene knockdown by being transcribed endogenously within cells from DNA vectors or engineered carriers. Once expressed, shRNA is processed into siRNA-like duplexes by the endogenous Dicer complex and incorporated into the RISC, leading to long-term silencing of target mRNAs [65]. This strategy is particularly advantageous in chronic diseases like OA, where prolonged modulation of pathogenic genes is essential for tissue regeneration and inflammation control [66]. Moreover, integrating shRNA with biologically compatible delivery vehicles, such as exosomes or hydrogels, can enhance the specificity, biosafety, and tissue retention of therapeutic RNA. A compelling example of this is the epigenetic regulation of EZH2, a histone methyltransferase known to suppress SOX9 expression, a master transcription factor for chondrogenesis, through H3K27me3-mediated chromatin silencing [67,68]. Targeting EZH2 via shRNA not only promotes ECM synthesis but also mitigates inflammatory pathways such as NF-κB, which are central to OA progression [69]. Building upon this rationale, Lv and colleagues developed an injectable multifunctional hydrogel, Exo^shEZH2^@HG, for cartilage repair (Fig. 2) [39]. The hydrogel was prepared by encapsulating shRNA-EZH2 endogenously modified exosomes (derived from bone mesenchymal stem cells (BMSCs)) into a photocrosslinkable hyaluronic acid (HA)/gelatin matrix [39]. The engineered exosomes delivered siRNA-EZH2 to target cells, effectively knocking down the EZH2 gene, which epigenetically silences SOX9, a key regulator of chondrogenesis [39]. This approach reduced H3K27me3-mediated repression of SOX9, promoting chondrocyte differentiation and ECM regeneration while inhibiting NF-κB-driven inflammation and apoptosis [39]. In a rat cartilage defect model, the hydrogel demonstrated sustained exosome release, enhanced cartilage regeneration, and prevented OA progression [39]. The study highlights the advantage of shRNA-based endogenous modification for precise, stable, and safe gene regulation, offering a synergistic strategy for tissue repair.

**Fig. 3:**
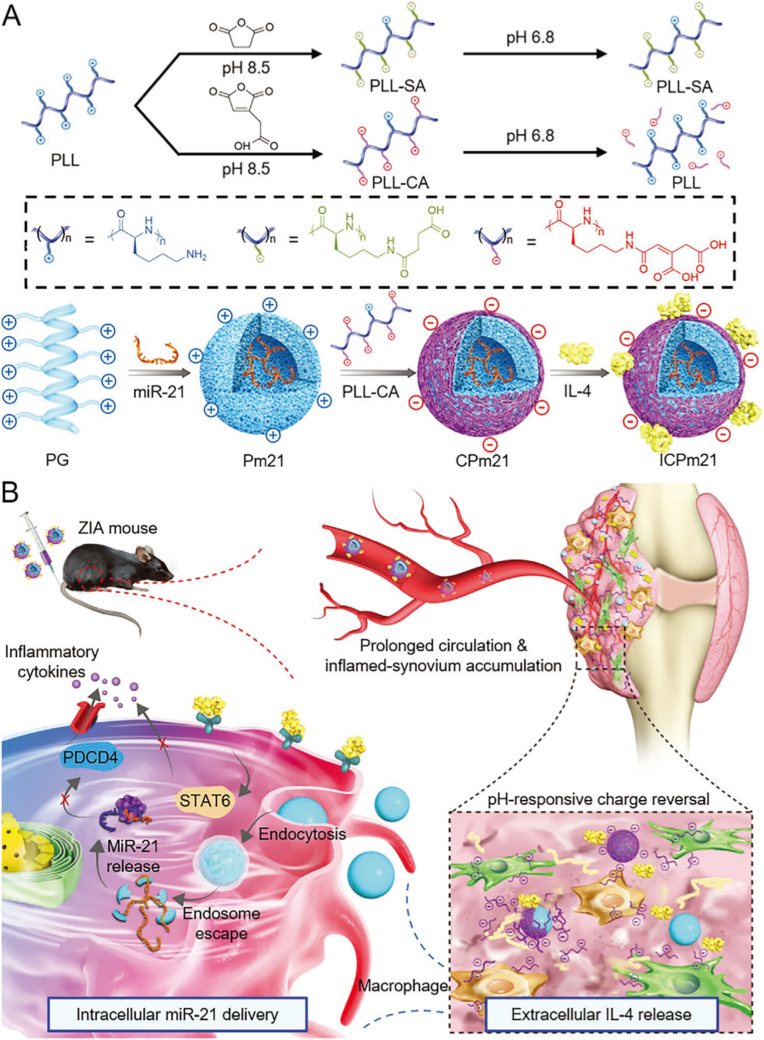
Schematic illustration showing the inflammation-instructed, hierarchical co-delivery of miR-21 and IL-4 mediated by acid-responsive, charge reversal NCs. Reproduced with permission from Ref. [43]. Copyright 2021 Wiley-VCH GmbH.

Compared to siRNA and shRNA, which typically target a single mRNA, miRNAs act as endogenous, pleiotropic regulators capable of fine-tuning multiple gene networks simultaneously. As part of the RISC, miRNAs bind to partially complementary sequences in the 3′ untranslated regions (3′ UTRs) of numerous mRNAs, leading to transcript degradation or translational repression [70,71]. This unique feature endows miRNAs with the ability to coordinate entire signaling cascades, making them ideal candidates for modulating complex inflammatory responses in diseases such as RA. Among the numerous miRNAs implicated in RA pathogenesis, microRNA-21 (miR-21) has emerged as a key immunoregulatory molecule that promotes macrophage polarization toward anti-inflammatory phenotypes by targeting pro-inflammatory genes such as PDCD4, a known activator of the NF-κB pathway [[72], [73], [74]]. Leveraging this insight, researchers have sought to co-opt miR-21 for therapeutic intervention, especially through responsive delivery platforms that ensure context-specific release in the inflammatory microenvironment. To this end, Deng et al. developed an inflammation-responsive nanocomplex (NC) for hierarchical co-delivery of IL-4 and miR-21 to treat RA (Fig. 3) [43]. The NC consisted of a cationic helical polypeptide (PG)/miR-21 core, coated with a pH-sensitive charge-reversal polymer (PLL-CA) and surface-adsorbed IL-4 [43]. Upon reaching the acidic synovial microenvironment, PLL-CA shed to release IL-4 extracellularly, while the exposed PG/miR-21 core efficiently transfected macrophages intracellularly [43]. MiR-21 silenced PDCD4 to inhibit NF-κB and polarize macrophages to the anti-inflammatory M2c phenotype, synergizing with IL-4-induced M2a polarization [43]. This dual-action NC attenuated inflammation, promoted resolution, and restored joint function in a murine RA model [43], demonstrating miRNA technology's precision in modulating immune responses and enhancing therapeutic outcomes.

While therapeutic delivery of miRNAs holds promise for reprogramming inflammatory microenvironments, dysregulated endogenous miRNAs are often themselves drivers of disease pathology, particularly in degenerative joint disorders [75]. In such contexts, anti-miRNA strategies, including antagomirs, have emerged as a powerful means to selectively inhibit pathological miRNA activity. Antagomirs are chemically modified, single-stranded oligonucleotides designed to bind with high specificity to complementary miRNAs, thereby blocking their interaction with target mRNAs [76]. This approach offers greater selectivity and minimal off-target effects, enabling targeted reversal of miRNA-mediated gene silencing. One compelling example is microRNA-365 (miR-365), which has been implicated in post-traumatic osteoarthritis (PTOA) by promoting pro-inflammatory signaling and ECM degradation via targets such as matrix metalloproteinase 13 (MMP13) and pro-inflammatory cytokines (e.g., IL-1β, TNF-α) [77]. To exploit this pathogenic axis, Zhang et al. developed a yeast cell wall particle (YCWP)-mediated nanotube-RNA delivery system loaded with miR-365 antagomir for PTOA therapy via oral administration [51]. The system was constructed by self-assembling non-toxic AAT nanotubes with miR-365 antagomir into NPs, which were encapsulated within YCWPs to withstand gastrointestinal degradation [51]. The YCWPs facilitated macrophage-specific uptake, enabling targeted delivery to inflamed joints. The anti-miR-365 approach effectively silenced miR-365, downregulating pro-inflammatory cytokines (IL-1β, IL-6, TNF-α) and MMP13, while upregulating protective factors (Nr1D2) [51]. This strategy demonstrated significant cartilage preservation and reduced inflammation in a murine PTOA model [51], highlighting the advantages of anti-miRNA technology, including high specificity, minimal off-target effects, and potential for oral gene therapy in degenerative diseases.

In contrast to antagomirs that silence deleterious miRNAs, mRNA therapies deliver exogenous transcripts encoding therapeutic proteins, enabling rapid and controlled protein production in target cells and offering a precise, transient approach to modulate gene expression [36]. Importantly, mRNA technologies enable cell-type-specific and antigen-specific modifications, making them particularly attractive for AIDs, such as RA, where restoring immune tolerance without broad immunosuppression is crucial. One key strategy involves reprogramming antigen-presenting cells (APCs) into a tolerogenic phenotype (tol-APCs), capable of suppressing autoreactive T cell responses and promoting regulatory T cell (Treg) expansion [[78], [79], [80]]. To address this, Liu et al. developed a low-immunogenicity LNP formulation, termed LNPs/mPDL1, to deliver mRNA encoding programmed death ligand 1 (PDL1) for generating tolerogenic antigen-presenting cells (tol-APCs) *in vivo* [52]. The LNPs were optimized using a design-of-experiment approach to minimize immunogenicity by adjusting the N/P ratio and lipid composition [52]. Upon subcutaneous injection, LNPs/mPDL1 selectively targeted APCs, inducing PDL1 expression to suppress overactivated T cells via the PDL1/PD1 pathway while sparing naive T cells [52]. In mouse models of RA and ulcerative colitis (UC), this approach reduced inflammation, promoted Treg expansion, and effectively halted disease progression [52]. The study highlights mRNA technology's advantages, including transient expression, low mutagenesis risk, and potential for antigen-specific modifications, offering a promising alternative to costly *ex vivo* tol-APC therapies for AIDs.

In summary, RNA-based therapies offer a versatile and precise platform for modulating gene expression and immune responses in autoimmune and inflammatory arthritides. siRNA/shRNA enables stable and specific gene silencing through RNAi, offering epigenetic reprogramming, as exemplified by the EZH2-targeting hydrogel system for cartilage repair [39]. miRNA mimics, such as miR-21, fine-tune immune pathways by suppressing pro-inflammatory signaling (e.g., NF-κB), thereby orchestrating macrophage polarization and joint protection in RA [43]. Conversely, anti-miRNA (antagomir) approaches block pathological miRNAs with high specificity and minimal off-target effects, as demonstrated by the oral delivery of miR-365 antagomir to mitigate cartilage degradation and inflammation in PTOA [51]. Lastly, mRNA therapies drive transient yet effective expression of therapeutic proteins, such as PDL1, to induce tolerogenic immune cell phenotypes *in vivo*, bypassing the need for *ex vivo* manipulation [52]. Collectively, these RNA modalities provide tailored, target-specific, and clinically translatable strategies, highlighting their transformative potential in next-generation immunotherapies.

### 2.2 DNA-based gene therapy

DNA-based therapies, particularly those employing plasmid DNA (pDNA), offer a promising and versatile approach for the treatment of arthritis. Unlike protein-based biologics that often suffer from short half-lives, high production costs, and the need for repeated administration, pDNA enables sustained, in situ expression of therapeutic proteins following a single administration. This allows for long-term modulation of disease-relevant pathways while minimizing systemic exposure and immune-related side effects [37]. In the context of arthritis, pDNA can be engineered to encode anti-inflammatory cytokines (e.g., IL-10, IL-1Ra) or regenerative factors (e.g., FGF18, BMPs) to actively suppress joint inflammation and promote cartilage repair [81,82]. These genetic payloads can be delivered locally, via intra-articular injection, to ensure targeted gene expression within affected tissues such as synovium or cartilage. Moreover, pDNA therapy avoids the risks of genomic integration associated with viral vectors, making it a safer option for repeated or long-term applications [83].

Beyond their safety and flexibility, pDNA therapies offer a strategic advantage in arthritis treatment by enabling controlled, localized expression of therapeutic genes directly within diseased joints. This approach allows the modulation of both inflammatory and degenerative processes through sustained production of anti-inflammatory cytokines or regenerative proteins, overcoming the limitations of recombinant protein delivery such as short half-life and systemic toxicity. Recent advances have focused on integrating pDNA into tissue-responsive biomaterials or cell-derived vesicles to further enhance joint retention, targeting efficiency, and therapeutic efficacy in both OA and RA. For instance, Wang et al. developed a stem cell-homing hydrogel system to deliver Klotho-expressing pDNA in combination with Tanshinone IIA for OA therapy [55]. Klotho, a known anti-aging and cartilage-protective factor, was locally expressed in damaged cartilage following intra-articular injection, promoting ECM restoration, reducing inflammation, and attenuating disease progression [84,85]. In addition, other studies have explored immune modulation in RA using similar strategies. Li et al. constructed M2 macrophage-derived exosome-based NPs to co-deliver IL-10 pDNA and betamethasone phosphate for synergistic inflammation control in RA [56]. This system not only enhanced IL-10 expression in inflamed joints but also induced macrophage repolarization toward an anti-inflammatory M2 phenotype, leading to effective suppression of synovial inflammation and joint destruction [56].

**Fig. 4:**
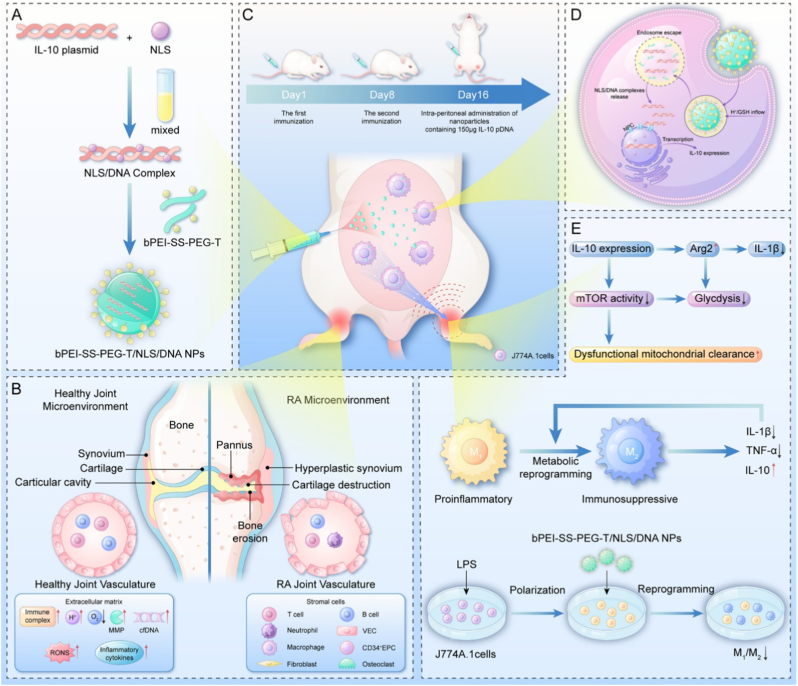
Schematic illustration of the macrophage-hitchhiking IL-10 pDNA delivery system against rheumatoid arthritis. Reproduced with permission from Ref. [54]. Copyright 2023 Published by Elsevier Ltd.

Building upon the therapeutic advantages of IL-10 pDNA in modulating immune responses, further research has focused on enhancing delivery precision and cellular specificity to maximize anti-inflammatory efficacy while minimizing systemic side effects. In particular, leveraging the natural inflammatory tropism of immune cells such as macrophages has emerged as an effective strategy to improve the targeted delivery of genetic payloads in RA. For example, Zhang et al. engineered a macrophage-hitchhiking nanoparticle system (bPEI-SS-PEG-T/NLS/DNA NPs) to treat RA by delivering IL-10 pDNA (Fig. 4) [54]. The NPs were prepared by electrostatic complexation of NLS peptides and IL-10 pDNA, followed by coating with tuftsin-modified disulfide-crosslinked polymers to enhance macrophage targeting and redox-responsive release [54]. Utilizing macrophages’ innate homing ability to inflamed joints, the system enabled targeted, intraperitoneal delivery [54]. Leveraging pDNA allowed sustained IL-10 expression, overcoming the rapid clearance of recombinant proteins [54]. Mechanistically, IL-10 pDNA reprogrammed M1 macrophages toward an anti-inflammatory M2 phenotype via mTOR pathway inhibition and Arg2 induction, leading to reduced TNF-α and IL-1β levels [54]. The system demonstrated low cytotoxicity, high transfection efficiency, and significant therapeutic effects in CIA rats, including decreased joint swelling and inflammation [54]. This work highlights the promise of pDNA-based therapies for achieving durable immune modulation in chronic inflammatory diseases like RA.

Beyond the use of pDNA to express anti-inflammatory cytokines or regenerative factors, DNA-based therapies have also been explored in the form of therapeutic DNA vaccines, particularly for autoimmune conditions such as RA. Unlike conventional protein vaccines or systemic immunosuppressants, DNA vaccines offer a safer, more precise approach by encoding disease-relevant autoantigens to induce antigen-specific immune tolerance, thereby selectively suppressing pathological immune responses while preserving normal immune function [86]. Their advantages include non-integration into the host genome, sustained yet controllable antigen expression, and relatively low immunogenicity of the plasmid vector compared to viral vectors [87]. Additionally, DNA vaccines are stable, cost-effective, and amenable to large-scale production, making them attractive for long-term management of chronic diseases. A representative example of this strategy is the study by Zhao et al., who developed a therapeutic DNA vaccine, pcDNA-CCOL2A1, encoding type II collagen (CII) for RA treatment [57]. The vaccine was constructed using the eukaryotic pcDNA3.1(+) vector, produced in *E. coli*, and purified for intramuscular or intravenous delivery [57]. The pDNA vaccine functions by transiently expressing CII mRNA and protein, which peaks within hours (2–6 h in blood) and clears within weeks (undetectable by day 42), as confirmed by RT-qPCR and bioluminescence imaging [57]. Importantly, the vaccine showed no genomic integration, ensuring safety. DNA vaccines offer advantages over traditional therapies, including avoiding generalized immunosuppression, inducing antigen-specific immune tolerance, and cost-effective production [57]. Preclinical results demonstrated that pcDNA-CCOL2A1 exhibited efficacy comparable to MTX in RA models, supporting its potential as a safe and targeted immunotherapy [88]. This study highlights the promise of DNA vaccines for AIDs like RA.

In conclusion DNA-based therapies offer a versatile and safe approach for the treatment of arthritis by enabling localized, sustained, and programmable expression of therapeutic genes. pDNA can encode anti-inflammatory cytokines (e.g., IL-10), regenerative factors (e.g., Klotho, FGF18), or disease-relevant autoantigens (e.g., CII) to achieve immune modulation, tissue repair, or immune tolerance. These therapies avoid genomic integration, reduce systemic side effects, and allow for flexible design tailored to disease mechanisms. Whether delivered via NPs, hydrogels, exosomes, or as DNA vaccines, pDNA-based interventions have demonstrated significant preclinical efficacy in both RA and OA models, highlighting their promise as a next-generation treatment platform for chronic inflammatory joint diseases.

### 2.3 CRISPR-Cas9-based gene therapy

CRISPR-Cas9 is a revolutionary genome-editing technology that enables precise, efficient, and programmable modification of specific DNA sequences. By utilizing a guide RNA to direct the Cas9 endonuclease to a targeted genomic site, CRISPR-Cas9 can induce double-strand breaks, leading to gene disruption or precise sequence correction through cellular repair mechanisms [38]. In the context of arthritis, CRISPR-Cas9 offers an unprecedented opportunity to intervene directly at the genetic level to modulate disease-driving pathways. Recent studies have explored CRISPR-Cas9-based strategies to silence pro-inflammatory genes such as TNF-α, IL-1β, and IL-6, thereby attenuating synovial inflammation and preventing joint destruction [12,89]. Beyond inflammation modulation, CRISPR-Cas9 has also been applied to promote cartilage regeneration by targeting key regulators of chondrocyte proliferation and matrix synthesis [90,91]. These approaches aim not only to halt disease progression but also to facilitate tissue repair, addressing the root causes of cartilage loss in arthritis. Compared to traditional therapies, CRISPR-Cas9-mediated editing provides highly specific, durable, and potentially curative effects with minimal off-target impact when properly optimized. Its flexibility allows for the simultaneous modulation of multiple pathogenic genes, offering a powerful tool for comprehensive arthritis management and the development of personalized gene therapies [92].

Targeting key pathogenic mediators at the genetic level is a crucial strategy for effective arthritis therapy. Several studies have leveraged CRISPR-Cas9 technology to directly edit genes involved in inflammation, cartilage degradation, and immune dysregulation. For instance, Zhao et al. used CRISPR-Cas9 to simultaneously disrupt NGF, MMP13, and IL-1β in OA models, significantly reducing inflammation and protecting cartilage integrity [12]. In RA, Li et al. applied CRISPR-Cas9 to intervene in B cell function by editing genes critical for B cell survival, achieving a marked decrease in B cell numbers and alleviation of autoimmune pathology [59]. Additionally, Dooley and Murphy utilized CRISPR-Cas9 to investigate the role of IL-16 in OA, uncovering its contribution to disease progression and providing a novel target for therapeutic intervention [89]. These findings underscore the potential of gene-specific editing strategies to address both inflammatory and degenerative mechanisms underlying arthritis.

**Fig. 5:**
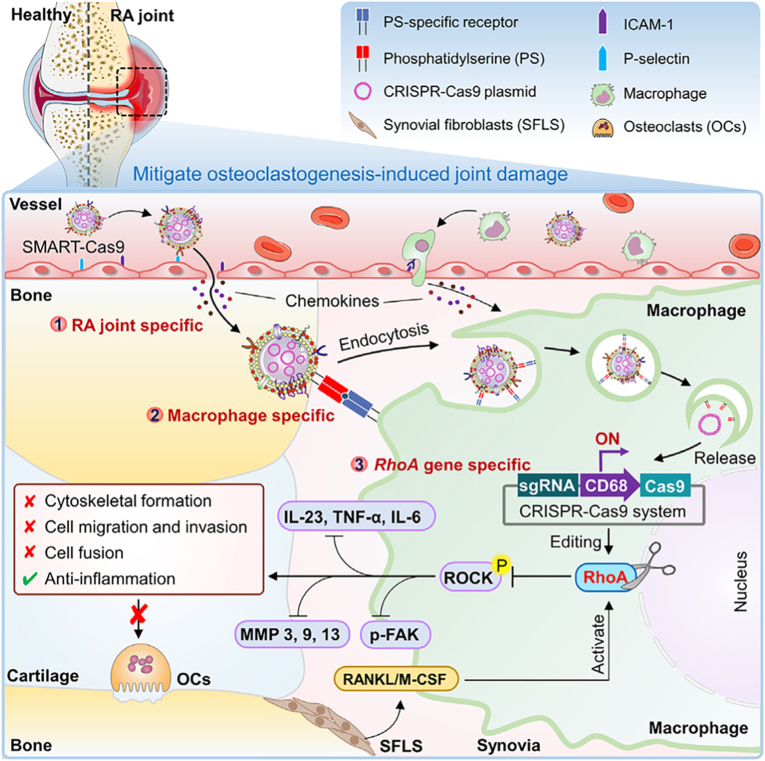
The schematic diagram of specific macrophage RhoA targeting CRISPR-Cas9 for mitigating osteoclastogenesis-induced joint damage in inflammatory arthritis. Reproduced with permission from Ref. [33]. Copyright 2025 Published by Elsevier Inc.

While CRISPR-Cas9 holds significant promise for arthritis therapy, its clinical translation heavily relies on the development of delivery systems that ensure cell specificity, minimize off-target effects, and achieve efficient gene editing in inflamed tissues [93]. Recent studies have focused on engineering targeted CRISPR delivery platforms to enhance therapeutic precision. For example, Chen et al. developed a macrophage-targeted CRISPR-Cas9 nanosystem, termed SMART-Cas9, to mitigate osteoclastogenesis-induced joint damage in RA (Fig. 5). [33]. The nanosystem was engineered by encapsulating RhoA-targeting CRISPR-Cas9 plasmids (driven by the macrophage-specific CD68 promoter) within phosphatidylserine (PS)-enriched macrophage membrane vesicles [33]. This design enabled precise delivery to RA joint macrophages, leveraging PS for enhanced phagocytosis and the CD68 promoter for cell-specific gene editing [33]. SMART-Cas9 effectively knocked out RhoA, disrupting cytoskeletal dynamics and osteoclast differentiation, thereby reducing bone erosion and inflammation in a CIA mouse model [33]. The study highlights CRISPR-Cas9's potential for cell-specific gene therapy, offering a targeted approach to treat inflammatory bone diseases with minimal off-target effects.

Beyond targeting macrophages to suppress osteoclastogenesis, CRISPR-Cas9 technology has also been harnessed to directly protect cartilage by editing pathogenic genes within chondrocytes. Given the central role of chondrocyte dysfunction and matrix degradation in OA, developing delivery systems that achieve efficient, selective gene editing in chondrocytes is critical for disease modification. For example, Liang et al. developed a chondrocyte-targeting hybrid exosome (hybrid CAP-Exo) for CRISPR-Cas9 plasmid delivery to treat OA [58]. The hybrid CAP-Exo was engineered by fusing liposomes with exosomes displaying a chondrocyte-affinity peptide (CAP) on their surface via genetic modification of the exosomal protein Lamp2b [58]. This hybrid system combined the targeting capability of exosomes with the high plasmid-loading capacity of liposomes [58]. Intra-articular injection of hybrid CAP-Exo encapsulating Cas9 sgMMP13 efficiently penetrated cartilage, selectively delivered the CRISPR-Cas9 system to chondrocytes, and knocked down MMP13, a key enzyme in cartilage degradation [58]. In OA rat models, this approach significantly reduced cartilage damage and restored ECM integrity [58]. The study highlights CRISPR-Cas9's precision in targeting disease-causing genes and demonstrates the potential of hybrid exosomes for safe, cell-specific gene editing therapies.

**Fig. 6:**
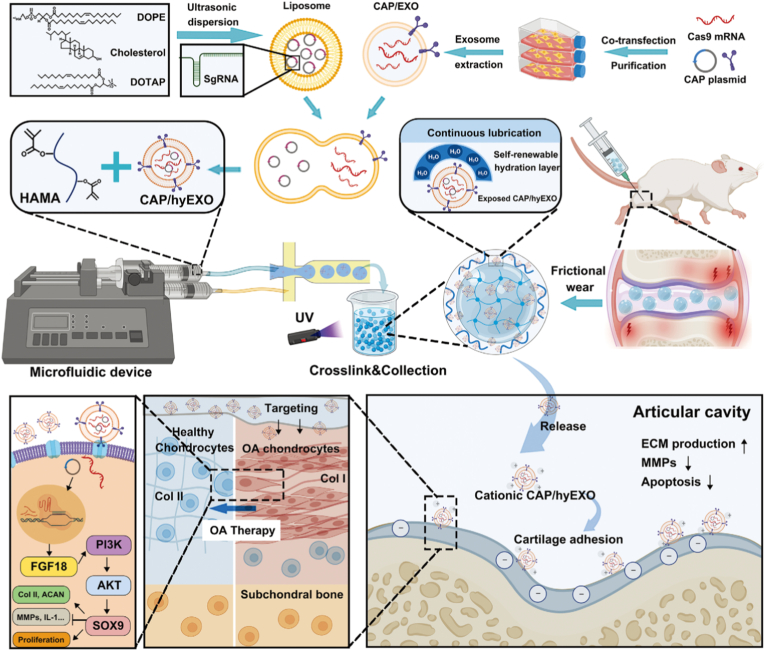
The development of an injectable CAP/FGF18-hyEXO@HMs system that combines with chondrocyte-targeted *in vivo* FGF18 gene-editing and self-renewable lubrication towards a cell-free OA treatment. Reproduced with permission from Ref. [60]. Copyright 2024 Wiley-VCH GmbH.

While activating anabolic pathways in chondrocytes through genome editing represents a powerful strategy for promoting cartilage repair, the combination of CRISPR-Cas9 with advanced biomaterials also opens new possibilities for enhancing therapeutic precision and durability. Beyond targeting FGF18, other studies have explored different molecular targets and delivery strategies to further optimize gene editing for OA treatment [90,91]. For example, Chen et al. developed an injectable microgel system, CAP/FGF18-hyEXO@HMs, for OA therapy (Fig. 6) [60]. The nanoplatform combines CRISPR-Cas9-based FGF18 gene-editing (delivered via chondrocyte-targeted hybrid exosomes, CAP/FGF18-hyEXO) with self-renewable lubrication (methacrylated hyaluronic acid microspheres, HMs) [60]. The hybrid exosomes were engineered by fusing liposomes (loaded with sgFGF18 plasmids) and exosomes (expressing Cas9 and a CAP), enabling precise genome-level FGF18 activation in OA chondrocytes [60]. The microgels ensured sustained release and joint lubrication [60]. Therapeutic outcomes included enhanced cartilage regeneration, reduced inflammation, and ECM degradation *in vitro* and *in vivo*, demonstrating the potential of CRISPR-Cas9 for long-term OA treatment by overcoming protein drug limitations (short half-life, repeated injections) [60]. This approach highlights the synergy of gene editing and biomaterials for targeted, durable therapies.

To sum up, CRISPR-Cas9 technology has emerged as a powerful tool for the treatment of arthritis by enabling precise, gene-specific interventions that directly target the molecular drivers of inflammation, cartilage degradation, and immune dysregulation. Through gene disruption or activation, CRISPR-Cas9 can silence pro-inflammatory cytokines such as TNF-α, IL-1β, and IL-6 [12,89], inhibit cartilage-destructive enzymes like MMP13 [58], or promote regenerative pathways such as FGF18 expression [60]. These interventions not only attenuate disease progression but also offer the potential for long-term tissue repair and functional restoration. Collectively, CRISPR-Cas9-mediated gene editing represents a transformative approach for arthritis therapy, with the potential to shift treatment paradigms from symptom management to disease modification and eventual resolution. Continued optimization of delivery strategies and rigorous assessment of safety will be essential to translating this technology into clinical practice.

## 3 Nanoplatforms for gene delivery

Efficient and targeted delivery remains one of the major challenges in the clinical translation of gene-based therapies, particularly for treating complex diseases such as arthritis. Naked nucleic acids, including siRNA, pDNA, or CRISPR-Cas9 components, are inherently unstable in biological environments and face barriers such as rapid degradation, poor cellular uptake, and nonspecific biodistribution [94]. To overcome these limitations, a wide range of nanoplatforms have been engineered to enhance gene delivery by improving stability, targeting, biocompatibility, and transfection efficiency. These delivery systems include lipid-based NPs, which are widely used due to their membrane fusion capabilities and clinical precedents; PNPs, offering tunable physicochemical properties and controlled release; and inorganic NPs, which provide structural rigidity and imaging potential. In addition, cell-derived vesicles and biomimetic membrane-coated systems leverage natural targeting and immune evasion features, while emerging designs such as tetrahedral framework nucleic acids (tFNAs) offer programmable and multifunctional architectures for precise cargo delivery. In the following sections, we summarize representative nanoplatforms used for delivering RNA, DNA, and CRISPR-Cas9 systems in arthritis models, highlighting their design strategies, biological performance, and therapeutic potential (Table 2).

**Table 2:** Nanoplatforms for gene delivery.

Nanoplatform	Formulations	Material composition/carrier structure	Type of genetic cargo	Refs
Lipid-based nanosystems	MTX + RELA siRNA FOL-Liposome	Liposomes with a CaP core composed of DSPC, and cholesterol.	siRNA-RELA	[41]
	PEG-Liposomal AntagomiR-155-5p	PEGylated liposomes composed of DPPC, cholesterol, and DSPE-PEG (62:35:3 M ratio).	Anti-miRNA-155-5p	[49]
	LNP@FAP siRNA	LNPs composed of NLS-SM102 (ionizable amino lipid), DMG-PEG-TAT (PEG polymer with targeting peptide), DSPC, and cholesterol.	siRNA-FAP	[95]
	mRNAb-LNPs	LNPs composed of ionizable lipid, cholesterol, DSPC, and DMG-PEG_2000_ (molar ratio 16:12:8:4).	mRNA-anti-CD19 antibody	[96]
Polymer nanoparticles	ITV-MT	Polymeric vesicles composed of tetra-mannose-functionalized PEG-P(TMC-DTC), and PEG-P(TMC-DTC)-PEI.	siRNA-TNF-α	[97]
	FP/miR-23b NPs	Fluorinated PAMAM dendrimer composed of modified with heptafluorobutyric anhydride.	miRNA-23b	[98]
Inorganic nanoplatforms	HIF-CaP-rHDL	CaP core coated with DOPA, encapsulated in apolipoprotein E3-reconstituted high-density lipoprotein.	siRNA-HIF-1α	[99]
	Urchin-like ceria NPs/miR-224-5p	Urchin-like CeO_2_ NPs (hydrothermal synthesis with Ce(NO_3_)_3_·6H_2_O and Na_3_PO_4_.	miRNA-224-5p	[100]
	CS-CDs@155	Ce/Se co-doped carbon dots (CS-CDs) with SOD-like (Ce) and GPx-like (Se) catalytic activity.	Cy5-labeled anti-miRNA-155	[50]
Extracellular vesicle-based nanosystems	Hybrid CAP-Exo/Cas9 sgMMP13	Engineered exosomes (CAP-Exo), displaying CAP on the Lamp2b surface protein, were fused with Lipofectamine 2000 liposomes to form hybrid CAP-Exo, which retained key exosomal markers (CD9, CD81, CD63).	CRISPR-Cas9 plasmid (Cas9 sgMMP13).	[58]
	M2 Exo/pDNA/BSP	Exosomes derived from M2-polarized macrophages; express anti-inflammatory markers (Arg-1, CD163).	pDNA-IL-10	[56]
	CAP-Exo/siMMP13	Native exosomes derived from ExpI293F cells; express exosomal markers (CD9, TSG101, Hsp70). Exo surface-modified with DSPE-PEG-MAL-conjugated CAP.	siRNA-MMP13	[101]
	CTP/Mir-EVs	EVs derived from hUCMSCs. EVs surface-modified with CII-targeting peptide (WYRGRL) fused to Lamp2b protein.	Exogenous miRNA-223 mimics	[102]
	OE-EVs	Engineered extracellular vesicles from RAW 264.7 macrophages overexpressing Lacc1 via plasmid transfection.	Exogenous Lacc1 gene (plasmid-based)	[103]
Membrane-coated nanoparticles	M2H@RPK	M2 macrophage membrane-coated NPs with HA-modified PEI complex (core: KAFAK-IRGD-PEI-shRNA-LEPR; shell: M2 membrane).	shRNA-LEPR	[29]
	MACP siTNF-α NPs	PB nanoenzymes coated with guanidinium-based polymers and macrophage membrane.	siRNA-TNF-α	[42]
	M-EHPS	EGCG-His-PBAP (EHP) complexed with siRNA via hydrogen bonding, electrostatic interactions, and hydrophobic forces, coated with M1 macrophage membrane.	siRNA-TNF-α	[104]
Tetrahedral DNA nanoframeworks	Tsi	tFNA composed of four ssDNA strands (ss1, ss2, ss3, ss4) self-assembled via Watson-Crick base pairing. siRNA-NF-κB is loaded onto tFNA via sticky-end binding.	siRNA-NF-κB	[105]
	T-23b	tFNA composed of four ssDNA strands (S1, S2, S3, S4) with miRNA-23b conjugated to the 3′ end of S4. Forms a tetrahedral nanostructure with a miRNA tail.	miRNA-23b	[106]
	T-(140 + 455)	tFNAs self-assembled from four ssDNA strands (S1-S4) with miRNA-140-5p and miRNA-455-3p conjugated to S2 and S3, respectively.	miRNA-140-5p and miRNA-455-3p	[107]

### 3.1 Lipid-based nanosystems

Lipid-based nanocarriers, including liposomes and LNPs, have emerged as leading platforms for gene delivery due to their excellent biocompatibility, low immunogenicity, and flexible physicochemical properties. These carriers are typically composed of one or more lipid components, such as ionizable lipids, phospholipids, cholesterol, and PEG-lipids, that self-assemble into bilayered vesicles or condensed nanostructures capable of encapsulating therapeutic nucleic acids [23,108]. Lipid-based systems offer several advantages for nucleic acid delivery. First, they protect vulnerable RNA and DNA molecules from nuclease degradation in the bloodstream. Second, they facilitate cellular uptake via endocytosis and enable endosomal escape, particularly through the use of ionizable lipids, which become protonated in acidic endosomes and destabilize the membrane. Third, they allow surface functionalization with targeting ligands, peptides, or antibodies to enhance tissue or cell specificity [20]. These features collectively improve pharmacokinetics, biodistribution, and transfection efficiency, which are key factors for successful gene therapy.

Mechanistically, LNPs typically encapsulate nucleic acids such as siRNA, mRNA, pDNA, or CRISPR-Cas9 components through electrostatic complexation and microfluidic-assisted self-assembly. Ionizable lipids carry a neutral charge at physiological pH, but gain a positive charge under acidic conditions, enabling efficient binding with negatively charged nucleic acids and minimizing systemic toxicity [20]. Lipid-based gene delivery has achieved remarkable clinical success, most notably with mRNA LNP vaccines (e.g., BNT162b2 and mRNA-1273 for COVID-19), which validated the scalability, safety, and efficacy of this approach. Building on this success, LNPs are now being actively investigated in clinical trials for liver-targeted siRNA therapies (e.g., Patisiran), mRNA-based cancer vaccines, and increasingly, chronic inflammatory diseases such as RA and OA, where localized and immune-responsive gene modulation is critical.

**Fig. 7:**
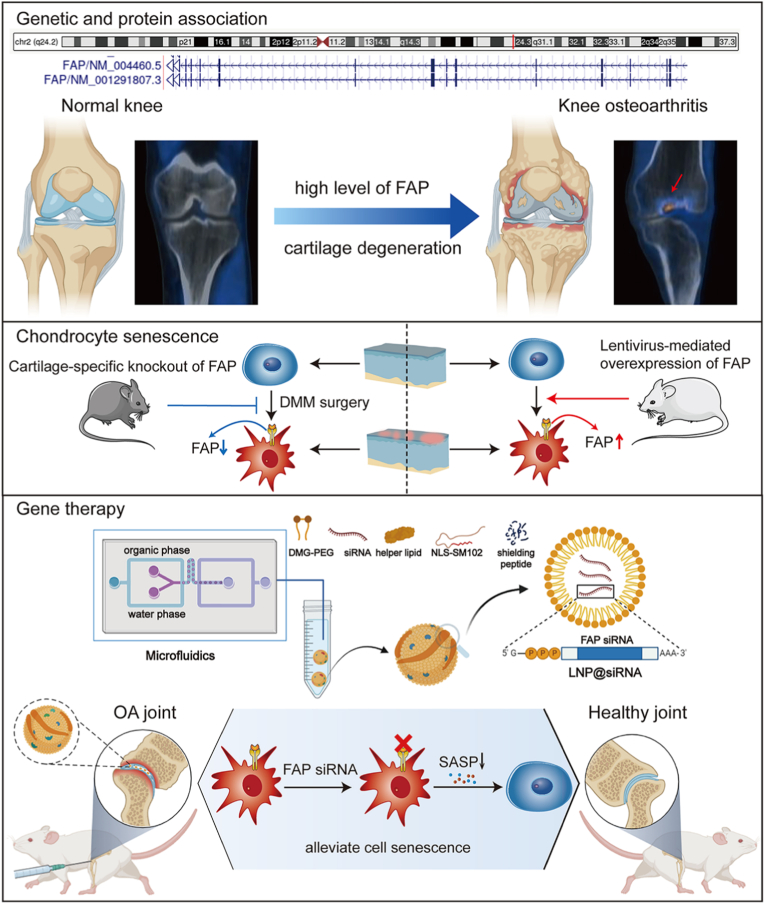
FAP-positive chondrocytes play a significant role in the pathogenesis of OA. Targeting these cells selectively has the potential to mitigate the progression of the disease. Our study provides valuable insights into the intraarticular injection of LNP@FAP siRNA as a promising strategy for the treatment of OA. Reproduced with permission from Ref. [95]. Copyright 2024 Springer Nature.

Among lipid-based platforms, LNPs have gained particular attention due to their compact structure, high *EE*%, and ability to mediate cytoplasmic delivery of nucleic acids without the need for viral vectors. For example, Guo et al. recently demonstrated that CD19-targeting mRNA encapsulated in LNPs showed therapeutic efficacy in preclinical models of lupus and rheumatoid arthritis, highlighting their potential for treating autoimmune diseases beyond conventional applications [96]. In addition, Zhao et al. developed a LNP-based siRNA delivery system targeting fibroblast activation protein (FAP) in chondrocytes to treat OA (Fig. 7) [95]. The LNPs were synthesized via microfluidics, incorporating ionizable lipids (NLS-SM102), PEG-modified targeting peptides (DMG-PEG-TAT), and FAP siRNA [95]. The optimized LNP@FAP siRNA exhibited a size of ∼74 nm, high stability, and efficient chondrocyte transfection [95]. Mechanistically, the LNPs delivered siRNA to silence FAP, inhibiting the NF-κB pathway and reducing senescence-associated secretory phenotype (SASP), thereby alleviating chondrocyte senescence and cartilage degeneration [95]. In a rat OA model, intra-articular LNP@FAP siRNA injections significantly reduced senescent cells, suppressed synovial inflammation, and preserved cartilage integrity [95], demonstrating its therapeutic potential for OA via targeted gene silencing.

In parallel, liposomes, as classical lipid bilayer vesicles, continue to serve as effective carriers for gene modulators, particularly in immunomodulatory applications. Their structural resemblance to cell membranes facilitates biocompatibility and fusion-mediated delivery, while surface modification enables immune cell targeting [109]. Paoletti et al. developed PEGylated liposomes (PEG-liposomes) encapsulating an antagonist targeting miRNA-155-5p (antagonist-155-5p) to treat RA [49]. The liposomes were prepared via a thin lipid film-hydration method using DPPC, cholesterol, and DSPE-PEG (62:35:3 M ratio), achieving ∼100 nm size and high *EE*% (>80 %) [49]. These liposomes selectively delivered antagonist-155-5p to monocytes/macrophages, silencing miRNA-155-5p and restoring anti-inflammatory macrophage (M2) polarization by upregulating SOCS-1 and C/EBP-β [49]. In murine RA models, intravenous PEG-liposome treatment reduced joint inflammation, decreased synovial immune cell infiltration, and improved arthritis scores without affecting other immune cells [49]. The study highlights PEG-liposomes as a targeted, safe strategy for RA therapy via myeloid-specific gene modulation. Consistently, Nasra et al. engineered folate-modified liposomes co-delivering methotrexate and RELA siRNA, which effectively reprogrammed macrophages, enhanced anti-inflammatory responses, and demonstrated synergistic therapeutic efficacy in both RAW264.7 cells and arthritic rat models, further underscoring the promise of liposome-based systems in rheumatoid arthritis treatment [41].

In short, lipid-based nanosystems represent a cornerstone of modern gene delivery strategies. Their advantages, such as biocompatibility, stability, and the ability to encapsulate and protect therapeutic nucleic acids, make them ideal for a wide range of applications, particularly in gene therapy and immunomodulation. These systems offer the flexibility to be surface-functionalized for targeted delivery, enhancing tissue specificity and improving therapeutic outcomes. As research advances, the potential for lipid-based carriers to treat complex diseases, including chronic inflammatory conditions, genetic disorders, and cancers, continues to grow. Their success in clinical trials, particularly in the realm of mRNA-based vaccines, sets a strong precedent for their future applications. Moving forward, the refinement of these systems will likely focus on optimizing their precision, minimizing off-target effects, and enhancing their long-term stability and efficacy *in vivo*.

### 3.2 Polymer nanoparticles

PNPs, including cationic block copolymer NPs, polymeric vesicles, and polyamidoamine (PAMAM) dendrimers, have emerged as promising carriers for nucleic acid-based therapies due to their excellent biocompatibility, adjustable properties, and high drug-loading capacities. These NPs are capable of effectively encapsulating nucleic acids such as DNA, and RNA, facilitating their delivery to target cells [21,22]. Cationic block copolymer NPs utilize electrostatic interactions to form stable complexes with nucleic acids, while polymeric vesicles offer unique properties for drug loading and controlled release, and PAMAM dendrimers provide high surface area and efficient drug encapsulation [110]. These polymeric systems are particularly valuable in gene therapy, RNAi, and mRNA vaccine development, where they can enhance the stability, targeted delivery, and cellular uptake of nucleic acid therapeutics. The future of PNPs in clinical applications holds significant potential to address key challenges in nucleic acid drug delivery, including stability, bioavailability, and targeted treatment outcomes [111].

**Fig. 8:**
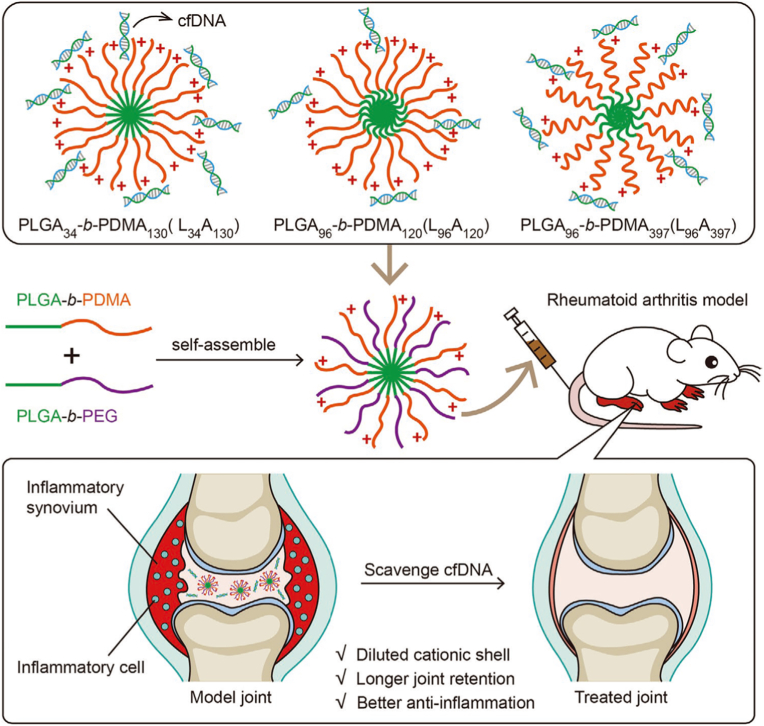
Binding cell-free DNAs that induce inflammation of rheumatoid arthritis with cationic nanoparticles may inhibit disease development. Toxicity of cationic nanoparticles from PLGA-b-PDMA may be compromised by incorporation of PEG segments to shell of nanoparticles to decrease charge density. Reproduced with permission from Ref. [114]. Copyright 2020 WILEY-VCH Verlag GmbH & Co. KGaA.

Building on the fundamental principles and mechanisms of PNPs, cationic block copolymer micelles (cNPs) represent a specific subclass that has garnered significant attention in the field of nucleic acid-based therapies and inflammatory disease treatments. These micelles are composed of amphiphilic block copolymers, where the cationic segment plays a crucial role in facilitating electrostatic interactions with negatively charged nucleic acids or other therapeutic agents. The versatility of cNPs lies in their ability to self-assemble or co-assemble into nanostructures in aqueous solutions, enabling tunable properties such as particle size, surface charge, and drug loading efficiency. The cationic nature of these micelles also allows for effective cellular uptake, making them an ideal carrier for gene delivery and anti-inflammatory treatments [112,113]. For example, Wu et al. developed cNPs composed of poly (lactic-co-glycolic acid)-block-poly(2-(dimethylamino)ethyl methacrylate) (PLGA-b-PDMA) with or without PEG segments for treating RA (Fig. 8) [114]. The cNPs were prepared via self-assembly or co-assembly of PLGA-b-PDMA and PLGA-b-PEG in aqueous solution, forming spherical NPs (∼40 nm) with tunable surface charge density [114]. These cNPs scavenged cell-free DNA (cfDNA) in inflamed joints through electrostatic interactions, thereby inhibiting cfDNA-induced inflammation [114]. PEGylation reduced cytotoxicity while enhancing joint accumulation and retention [114]. In a CIA rat model, optimized cNPs (e.g., PLGA34-b-PDMA130 with 50 % PEG) significantly alleviated joint swelling and tissue damage without systemic toxicity [114], demonstrating their potential as a targeted RA therapy.

Building upon the concept of cNPs, another significant advancement in PNP design comes from the development of inflammation-targeted polymeric vesicles (ITV-MT), as demonstrated by Liang Yang et al. [97]. This study addresses the challenge of co-delivering both small molecules and nucleic acids to targeted tissues, which is particularly relevant in the treatment of complex inflammatory diseases such as RA [97]. Unlike the more commonly studied cationic micelles, polymeric vesicles offer unique advantages in drug delivery due to their ability to encapsulate both hydrophobic and hydrophilic drugs simultaneously, providing a platform for multi-drug therapies. In their work, Liang and colleagues engineered ITV-MT vesicles for the co-delivery of MTX and siRNA-TNF-α (siTNFα) to treat RA [97]. These vesicles were prepared by co-self-assembly of tetra-mannose-functionalized PEG-P(TMC-DTC) and PEG-P(TMC-DTC)-PEI triblock copolymers, forming disulfide-crosslinked vesicles with a size of approximately 53 nm [97]. The vesicles exhibited a high drug-loading capacity (17.1 wt% MTX, 9.0 wt% siTNFα), which is crucial for the efficacy of combination therapies [97]. The tetra-mannose ligands on the vesicle surface played a key role in targeting macrophages via CD206 binding, a receptor that is upregulated on activated macrophages, while the PEI core facilitated the complexation of siRNA, ensuring effective gene silencing [97]. The combination of MTX, a widely used anti-inflammatory drug, and siTNFα, which targets a critical proinflammatory cytokine in RA, allowed ITV-MT to synergistically suppress proinflammatory cytokines (TNF-α, IL-6) and promote the repolarization of macrophages from the pro-inflammatory M1 phenotype to the anti-inflammatory M2 phenotype [97]. In a CIA mouse model, ITV-MT demonstrated enhanced joint accumulation, reduced paw swelling, and protection against bone erosion [97]. The results clearly indicated superior therapeutic efficacy compared to monotherapy with either MTX or siTNFα alone [97], emphasizing the potential of these targeted polymeric vesicles for co-delivering both small molecules and siRNA to achieve a more effective treatment outcome.

In addition, PAMAM dendrimers are a class of highly branched, spherical polymers with a unique nanoscale architecture that offers several distinct advantages in drug delivery systems, particularly in gene therapy. Their well-defined, tree-like structure provides a high degree of surface functionality, which can be easily modified to enhance biocompatibility, targeting capabilities, and drug loading efficiency [115]. Due to their versatile surface groups, PAMAM dendrimers can efficiently complex with nucleic acids, facilitating their delivery to target cells. Additionally, their nanoscale size allows for enhanced cellular uptake, and their multivalency enables interaction with multiple receptors or biomolecules, improving the efficacy of targeted therapies [115,116]. PAMAM dendrimers also offer the benefit of controlled release, allowing for the sustained delivery of therapeutic agents [116]. For example, Han et al. developed a fluorinated PAMAM (FP) to deliver microRNA-23b (miR-23b) for treating experimental RA in rats [98]. The FP was synthesized by modifying PAMAM with heptafluorobutyric anhydride, forming stable NPs (∼230 nm) at an N/P ratio of 2.0. FP enhanced miR-23b delivery via clathrin-mediated endocytosis and improved endosomal escape, restoring miR-23b levels in macrophages [98]. The NPs targeted TAB2, TAB3, and IKKα to inhibit NF-κB signaling, reducing pro-inflammatory cytokines (TNF, IL-6, IL-1β) and inducing apoptosis via caspase-9 activation [98]. In RA models, FP/miR-23b NPs accumulated in inflamed joints, alleviated synovitis, and reduced bone erosion with negligible systemic toxicity, demonstrating dual anti-inflammatory and pro-apoptotic effects [98]. This study highlights FP as an efficient non-viral gene carrier for RA therapy.

In conclusion, PNPs represent a transformative approach in the field of nucleic acid delivery, offering remarkable potential for advancing gene therapy and RNA-based interventions. With their customizable properties, such as size, charge, and drug-loading capacity, PNPs provide versatile platforms for effective encapsulation and targeted delivery of nucleic acids. These NPs address key challenges in therapeutic delivery, including stability, bioavailability, and controlled release, while also enabling efficient cellular uptake. Their unique architecture and surface functionalities allow for fine-tuned interactions with target cells, thereby enhancing the specificity and efficacy of treatment [110]. Looking ahead, the development of advanced PNP systems holds immense promise in revolutionizing the treatment of a wide range of diseases, particularly those with complex pathophysiologies such as AIDs and cancers. The continued refinement of PNP technologies, alongside innovations in targeting, multi-drug co-delivery, and precision medicine, will enable more effective and personalized therapeutic strategies. Moreover, the potential for scalable, reproducible production processes opens new avenues for translating these cutting-edge technologies into clinical practice. As research progresses, PNPs will undoubtedly play a central role in shaping the future of molecular medicine, offering unprecedented opportunities for overcoming current therapeutic limitations and improving patient outcomes.

### 3.3 Inorganic nanoplatforms

Inorganic NPs, such as ceria and calcium phosphate (CaP) NPs, have gained attention as effective carriers for gene delivery due to their excellent stability, biocompatibility, and customizable properties. These NPs provide a stable platform for encapsulating nucleic acids, protecting them from degradation in the body, and enhancing their delivery to target cells. Their high stability ensures that the genetic material remains intact until it reaches its destination, making them ideal for sustained therapeutic release [117,118]. One major advantage of inorganic NPs is their ability to be easily modified with various surface coatings, such as PEG or targeting ligands, allowing for better control over their size, surface charge, and drug loading capacity. This functionalization enables targeted delivery to specific cells, improving the efficiency of gene transfer while minimizing off-target effects [119]. Gene delivery using inorganic NPs generally relies on electrostatic interactions between the negatively charged nucleic acids and the positively charged nanoparticle surfaces. Once inside the target cells, the NPs can facilitate the release of genetic material, often by taking advantage of environmental changes like pH. This controlled release is essential for effective gene therapy, as it ensures that the genetic material reaches the desired location and is released in a controlled manner [117,120].

**Fig. 9:**
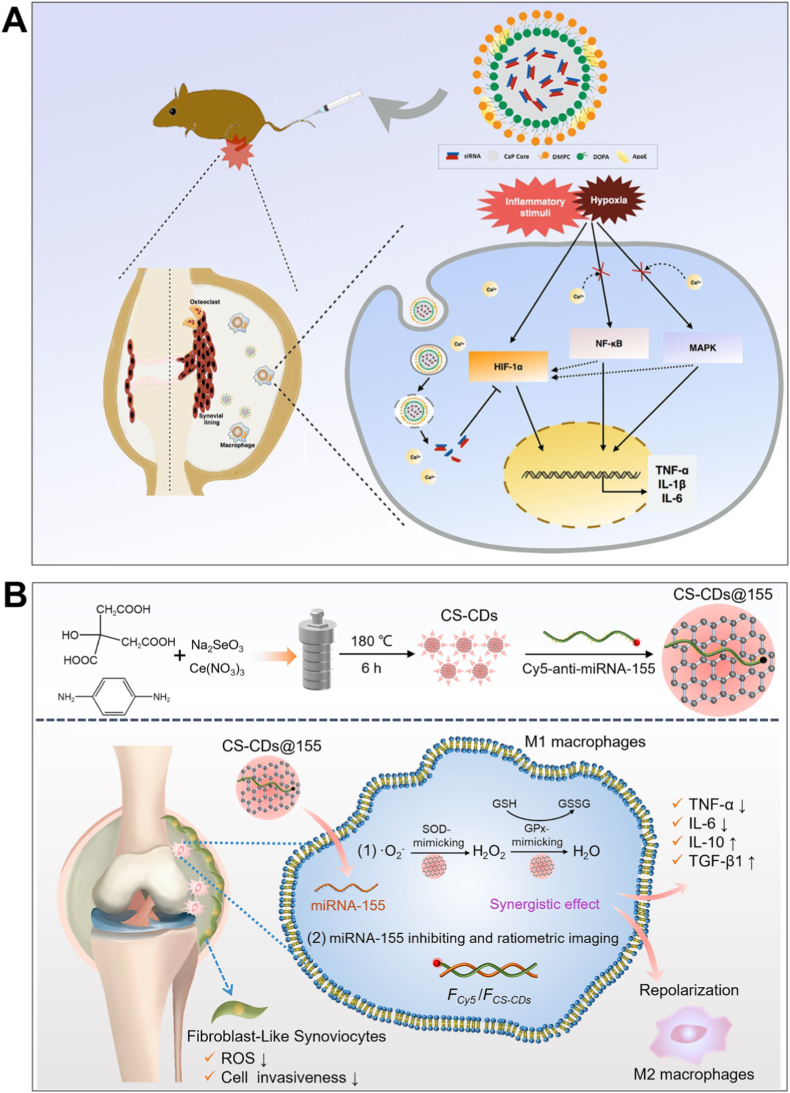
(A) Schematic illustration of CaP-based nanoparticles encapsulating HIF-1α siRNA for anti-inflammatory therapy in rheumatoid arthritis. Reproduced with permission from Ref. [99]. Copyright 2020 Elsevier B.V. (B) Schematic illustrating the production and use of CS-CDs/Cy5-*anti*-miRNA-155 (CS-CDs@155). Reproduced with permission from Ref. [50]. Copyright 2024 Elsevier B.V.

Inorganic NPs have gained significant attention in the field of gene delivery due to their stability, biocompatibility, and ability to encapsulate genetic materials effectively. Among these, CaP NPs stand out for their ability to encapsulate nucleic acids such as siRNA, DNA, and mRNA, making them a promising tool for targeted gene therapies. The unique properties of CaP NPs, including their ability to form stable complexes with genetic materials and their ability to degrade *in vivo* to release the payload, position them as a powerful system for gene silencing, particularly in inflammatory diseases like RA [121]. For example, Liu et al. developed a CaP-based NP encapsulating HIF-1α siRNA (HIF-CaP-rHDL) for RA therapy (Fig. 9A) [99]. The NPs were synthesized via a water-in-oil microemulsion method, forming CaP cores coated with dioleoylphosphatidic acid (DOPA) and further encapsulated in apolipoprotein E3-reconstituted high-density lipoprotein (rHDL) to enhance stability and targeting [99]. HIF-CaP-rHDL (∼80 nm) efficiently delivered siRNA to macrophages, silencing HIF-1α and inhibiting NF-κB and MAPK pathways, thereby reducing pro-inflammatory cytokines (TNF-α, IL-1β, IL-6) and osteoclastogenesis [99]. In CIA mice, systemic administration of HIF-CaP-rHDL accumulated in inflamed joints, alleviated synovitis, and mitigated bone erosion and cartilage damage [99]. The study highlights CaP-rHDL as a promising gene delivery system for RA by combining siRNA-mediated gene silencing with anti-inflammatory and anti-osteoclastogenic effects.

In addition, among the various inorganic nanoparticle systems, cerium oxide (ceria) NPs have emerged as a promising option due to their remarkable antioxidant properties, which can counteract oxidative damage in tissues [122]. Additionally, ceria NPs are known for their high surface area and the ability to efficiently encapsulate genetic materials, making them an ideal candidate for gene therapy applications. For example, Chen et al. developed urchin-like ceria NPs for enhanced gene therapy of OA [100]. The NPs were synthesized via hydrothermal reaction between Ce(NO_3_)_3_·6H_2_O, Na_3_PO_4_, and water, yielding a high surface area thorny structure rich in Ce^4+^, which conferred superior reactive oxygen species (ROS)-scavenging catalase-like activity [100]. These NPs efficiently delivered microRNA-224-5p (miR-224-5p) into chondrocytes via electrostatic adsorption and endocytosis, overcoming poor cellular uptake and stability limitations of free miRNA [100]. The NPs/miR-224-5p conjugate synergistically reduced oxidative stress, promoted autophagy, inhibited apoptosis, and protected cartilage ECM by downregulating catabolic mediators (e.g., MMP13) and inflammatory cytokines [100]. *In vivo*, intra-articular injection alleviated OA progression by mitigating synovitis, subchondral bone sclerosis, and joint space narrowing [100]. The study highlights ceria NPs as a dual-functional platform combining ROS scavenging and gene delivery for OA therapy.

Beyond their role as gene carriers, certain inorganic NPs possess intrinsic enzyme-mimicking properties, known as nanozyme activity. These nanozymes can catalyze biochemical reactions similarly to natural enzymes, particularly in pathological environments characterized by elevated oxidative stress, such as in RA [123]. By combining nucleic acid delivery with ROS-scavenging or redox-modulating capabilities, nanozyme-based platforms offer a dual-functional approach to gene therapy. This synergistic effect not only enhances the stability and cellular uptake of nucleic acids but also contributes directly to immune modulation and inflammation resolution [124]. In the context of RA, leveraging such platforms to both deliver gene regulators and reprogram inflammatory macrophages has emerged as a powerful strategy. For example, Gong et al. developed cerium (Ce) and selenium (Se) co-doped carbon dots (CS-CDs) as self-cascading antioxidant nanozymes for RA treatment (Fig. 9B) [50]. The CS-CDs were synthesized via a hydrothermal reaction using citric acid, p-phenylenediamine, cerium nitrate, and sodium selenite, resulting in NPs with dual enzyme-mimicking activities: Ce conferred superoxide dismutase (SOD)-like activity to scavenge superoxide radicals (•O_2_^−^), while Se provided glutathione peroxidase (GPx)-like activity to decompose H_2_O_2_, thereby mimicking the natural antioxidant defense system [50]. The CS-CDs were further loaded with Cy5-labeled anti-miRNA-155 (CS-CDs@155) to simultaneously monitor and regulate macrophage polarization [50]. *In vitro* and *in vivo* studies demonstrated that CS-CDs@155 effectively scavenged ROS, suppressed pro-inflammatory cytokines (e.g., TNF-α, IL-6), and promoted M1-to-M2 macrophage repolarization, alleviating joint inflammation and cartilage damage in RA models [50]. This nanozyme-based strategy achieved synergistic ROS elimination and anti-inflammatory effects, offering a promising approach for RA theranostics.

In conclusion, inorganic NPs, with their high stability, biocompatibility, and tunable surface characteristics, present a highly promising platform for gene delivery. These systems overcome many of the limitations associated with traditional delivery methods, such as poor stability and low specificity. With the ongoing advancements in nanoparticle engineering and surface functionalization, inorganic NPs hold great potential for advancing the field of gene therapy, RNAi, and other nucleic acid-based treatments. Their ability to efficiently deliver genetic material to target cells, along with the potential for controlled release and co-delivery of multiple therapeutic agents, positions them as a transformative tool in the development of next-generation therapies.

### 3.4 Extracellular vesicle-based nanosystems

Extracellular vesicle (EV)-based nanosystems represent a versatile and biomimetic platform for gene delivery, offering several intrinsic advantages over synthetic carriers [125,126]. As naturally secreted lipid bilayer vesicles, EVs possess excellent biocompatibility, low immunogenicity, and the inherent ability to evade phagocytic clearance and cross physiological barriers, such as the blood-brain barrier and dense extracellular matrices [58,127]. Their membrane composition, enriched with adhesion molecules and surface proteins, facilitates efficient cellular uptake through mechanisms like endocytosis, membrane fusion, or receptor-ligand interactions [128]. Moreover, EVs can encapsulate diverse nucleic acid cargos, including siRNA, miRNA, and plasmids, protecting them from enzymatic degradation in circulation [[129], [130], [131]]. Notably, EVs can be bioengineered through donor cell modification to enhance their targeting capabilities, loading efficiency, or therapeutic function. Such engineering strategies include the introduction of surface ligands, fusion proteins, or intracellular machinery to selectively package desired cargos. These modifications allow EVs to achieve active targeting, stimuli-responsive release, and precise modulation of recipient cell behavior [132]. Overall, EV-based nanosystems offer a biologically optimized and customizable approach for safe, effective, and targeted gene delivery.

**Fig. 10:**
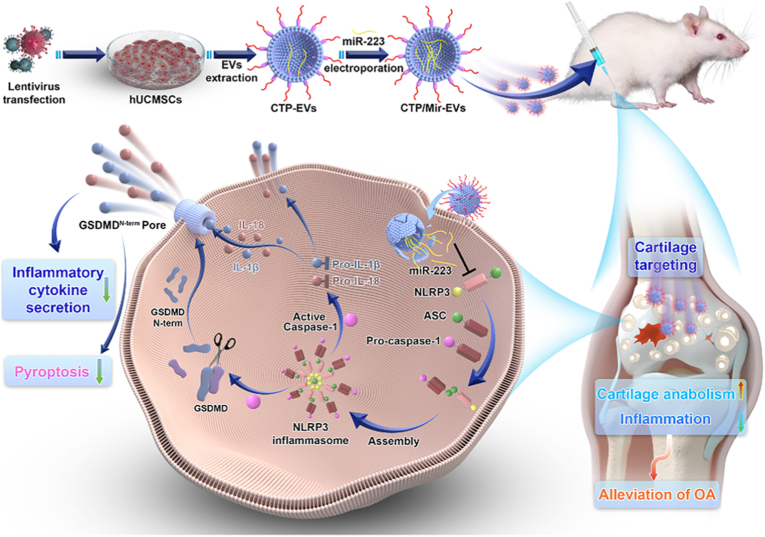
A schematic overview of dual-engineered cartilage-targeting extracellular vesicles derived from mesenchymal stem cells enhance osteoarthritis treatment via miR-223/NLRP3/pyroptosis axis: Toward a precision therapy. Reproduced with permission from Ref. [102]. Copyright 2023 KeAi Communications Co. Ltd.

Mesenchymal stem cell (MSC)-derived EVs have emerged as particularly attractive carriers for therapeutic nucleic acid delivery due to their innate regenerative and immunomodulatory properties. Among various sources, EVs derived from human umbilical cord mesenchymal stem cells (hUCMSCs) offer additional advantages such as low immunogenicity, ethical accessibility, and a high yield of secreted vesicles [133]. These EVs naturally carry a repertoire of bioactive molecules that contribute to tissue repair and inflammation resolution, making them especially suitable for diseases like OA, where cartilage degradation and chronic inflammation coexist. Furthermore, MSC-EVs exhibit strong tropism toward damaged tissues, and their lipid bilayer structure enables efficient encapsulation and protection of RNA therapeutics [134]. For example, Liu et al. developed a dual-engineered cartilage-targeting EV derived from hUCMSCs for OA treatment (Fig. 10) [102]. The EVs were modified by electroporation to load exogenous microRNA-233 (miR-223), which inhibits NLRP3 inflammasome activation, and genetically engineered with a CII-targeting peptide (WYRGRL) on their surface for enhanced cartilage specificity [102]. These engineered EVs (CTP/Mir-EVs) effectively delivered miR-223 to chondrocytes, suppressing NLRP3-mediated pyroptosis and inflammation while promoting cartilage anabolism [102]. *In vivo* studies demonstrated significant reductions in cartilage degradation and inflammatory markers in OA rat models, highlighting the potential of precision-engineered EVs as a therapeutic strategy for OA [102]. The study underscores the utility of EV-based delivery systems for targeted RNA therapy in degenerative joint diseases.

In addition to MSC-derived EVs, macrophage-derived EVs are also gaining attention as versatile carriers for nucleic acid and protein delivery in inflammatory and degenerative diseases. These vesicles inherit the immunomodulatory and targeting characteristics of their parental cells, enabling selective homing to inflamed or damaged tissues [135]. Moreover, macrophage-derived EVs can be genetically or chemically engineered to incorporate therapeutic agents or enhance functional specificity, offering a powerful strategy for disease-tailored interventions. For example, Hu et al. developed Lacc1-engineered extracellular vesicles (OE-EVs) derived from macrophages to treat temporomandibular joint osteoarthritis (TMJOA) [103]. The EVs were modified by transfecting RAW 264.7 macrophages with Lacc1-overexpressing plasmids, followed by isolation via ultracentrifugation [103]. OE-EVs delivered Lacc1 to chondrocytes, reprogramming their mitochondrial metabolism by inhibiting glycolysis, reducing ROS, and restoring oxidative phosphorylation (OXPHOS) [103]. This metabolic shift alleviated inflammation, suppressed catabolic factors (e.g., MMPs, IL-1β), and promoted cartilage matrix synthesis (e.g., CII, aggrecan) [103]. *In vivo*, OE-EVs enhanced cartilage repair, reduced subchondral bone erosion, and improved proteoglycan retention in TMJOA mice [103], demonstrating their potential as a targeted metabolic therapy for degenerative joint diseases.

Furthermore, exosomes, a subtype of EVs, offer distinct advantages for therapeutic delivery [136,137]. Compared to larger vesicles, exosomes exhibit superior biocompatibility, nanoscale size for deep tissue penetration, and natural stability in biological fluids. They possess inherent targeting capabilities derived from their parent cells and can cross biological barriers with minimal immunogenicity [138]. Their lipid bilayer structure enables the encapsulation of diverse therapeutic cargos, including nucleic acids, proteins, and small molecules [139]. For example, Li et al. developed M2 macrophage-derived exosomes (M2 Exo) co-loaded with IL-10 pDNA and betamethasone sodium phosphate (BSP) for RA therapy [56]. The exosomes were isolated from IL-4-polarized M2 macrophages, and IL-10 pDNA was transfected into M2 macrophages via lipofectamine, while BSP was electroporated into harvested exosomes [56]. The resulting M2 Exo/pDNA/BSP NPs (84–100 nm) exhibited inflammation-targeting via LFA-1/ICAM-1 interactions and intrinsic anti-inflammatory properties [56]. The system synergistically promoted M1-to-M2 macrophage repolarization by upregulating IL-10 and downregulating TNF-α/IL-1β [56]. In CIA mice, it reduced joint inflammation, cartilage erosion, and synovial hyperplasia with minimal toxicity [56], demonstrating dual gene/drug delivery efficacy.

Building upon the inherent advantages of exosomes, further functionalization of their surface can significantly enhance their therapeutic potential. Surface modification strategies, such as peptide conjugation, lipid insertion, or genetic engineering, allow for improved targeting specificity, prolonged circulation time, and better tissue penetration [138]. Notably, exosomes derived from ExpI293F cells offer advantages such as high yield, ease of genetic manipulation, and reduced immunogenicity, making them an ideal source for engineered vesicles [140,141]. These modifications enable exosomes to actively home to diseased tissues, increasing local drug concentration while minimizing off-target effects and systemic toxicity [138]. Such engineering approaches make exosomes a versatile and precise platform for delivering nucleic acid therapies and small molecules in complex disease settings. For example, Zhang et al. developed chondrocyte-targeted exosomes (CAP-Exo) for siRNA delivery to treat OA [101]. The exosomes, derived from ExpI293F cells, were surface-functionalized with a CAP via lipid insertion (DSPE-PEG-MAL-CAP). siRNA against matrix metallopeptidase 13 (siMMP13) was loaded into CAP-Exo by electroporation, yielding CAP-Exo/siMMP13 [101]. This nanoplatform demonstrated enhanced chondrocyte targeting and retention in joints, enabling efficient siRNA delivery [101]. *In vitro* and *in vivo* studies showed CAP-Exo/siMMP13 significantly suppressed MMP13 expression, upregulated collagen COL2A1, and restored cartilage matrix homeostasis, alleviating OA progression in a rat model [101]. The engineered exosomes achieved targeted gene silencing with minimal off-target effects, highlighting their potential for RNAi-based OA therapy.

Collectively, EV-based delivery systems have demonstrated remarkable potential in the treatment of OA and other inflammatory joint diseases. Leveraging their natural biocompatibility, membrane fusion capacity, and ability to cross biological barriers, EVs, particularly those derived from MSCs, macrophages, or ExpI293F cells, enable efficient and targeted delivery of therapeutic cargos such as miRNAs, siRNAs, peptides, and small-molecule drugs. Further engineering, including surface functionalization or genetic manipulation, enhances their targeting precision, cargo-loading efficiency, and disease-modifying capabilities. These strategies have been shown to modulate immune responses, reprogram cellular metabolism, and restore cartilage homeostasis *in vivo*. Looking forward, EV-based nanotherapeutics hold promise for clinical translation as next-generation, cell-free platforms offering personalized, minimally invasive, and highly specific therapies for degenerative and autoimmune joint diseases.

### 3.5 Membrane-coated nanoparticles

Membrane-coated nanoparticles (MCNPs) represent an emerging class of biomimetic delivery systems that combine the structural versatility of synthetic nanocarriers with the functional complexity of natural cell membranes. By camouflaging NPs with membranes derived from specific cell types, MCNPs inherit source-cell properties such as immune evasion, targeted adhesion, and biological communication, significantly enhancing their *in vivo* performance [142,143]. In the context of arthritis, membrane coating strategies, using membranes from macrophages, neutrophils, synoviocytes, or stem cells, have been shown to improve targeting to inflamed joints, prolong circulation time, and reduce off-target effects [144]. Among them, macrophage MCNPs are particularly advantageous due to their intrinsic inflammation-homing ability and immunomodulatory surface proteins. These coatings can actively target inflamed synovium via interactions with adhesion molecules (e.g., ICAM-1, VCAM-1) and sequester pro-inflammatory cytokines, thereby mitigating local inflammation and enhancing therapeutic efficacy [145]. This strategy offers a promising avenue for precise and sustained treatment of arthritis with improved biocompatibility and joint-specific delivery.

In OA, synovial inflammation and cartilage degradation are central to disease progression, and effective therapeutic strategies that can modulate these pathological processes are still lacking. The challenge lies in selectively targeting the inflamed tissues without causing systemic toxicity, while simultaneously promoting cartilage repair [146]. In this context, macrophage MCNPs have emerged as a promising class of biomimetic carriers that exploit the natural inflammation-homing properties of macrophages. Macrophages, especially those in an activated state, are known to accumulate at sites of inflammation, making them an ideal vehicle for targeted delivery. Additionally, these NPs possess immune-evasive capabilities due to the membrane's ability to mask the NP's foreign surface, which reduces clearance by the immune system, enhancing the NPs' circulation time and targeting efficiency [147,148]. For example, Zhou et al. developed an M2 macrophage membrane-coated nanoparticle system (M2H@RPK) for targeted OA therapy [29]. The NPs were prepared by condensing anti-inflammatory peptide KAFAK and shRNA-LEPR with polyethylenimine (PEI), modifying them with HA, and coating them with M2 macrophage membranes (M2M) [29]. The M2M coating enhanced synovial targeting and macrophage uptake, promoting M1-to-M2 repolarization to reduce pro-inflammatory cytokines (TNF-α, IL-1β) and elevate anti-inflammatory IL-10 [29]. In OA rats, M2H@RPK significantly alleviated synovitis, preserved cartilage integrity, and improved joint function [29], demonstrating its potential as a disease-modifying OA therapy through synergistic immune modulation and sustained drug release.

**Fig. 11:**
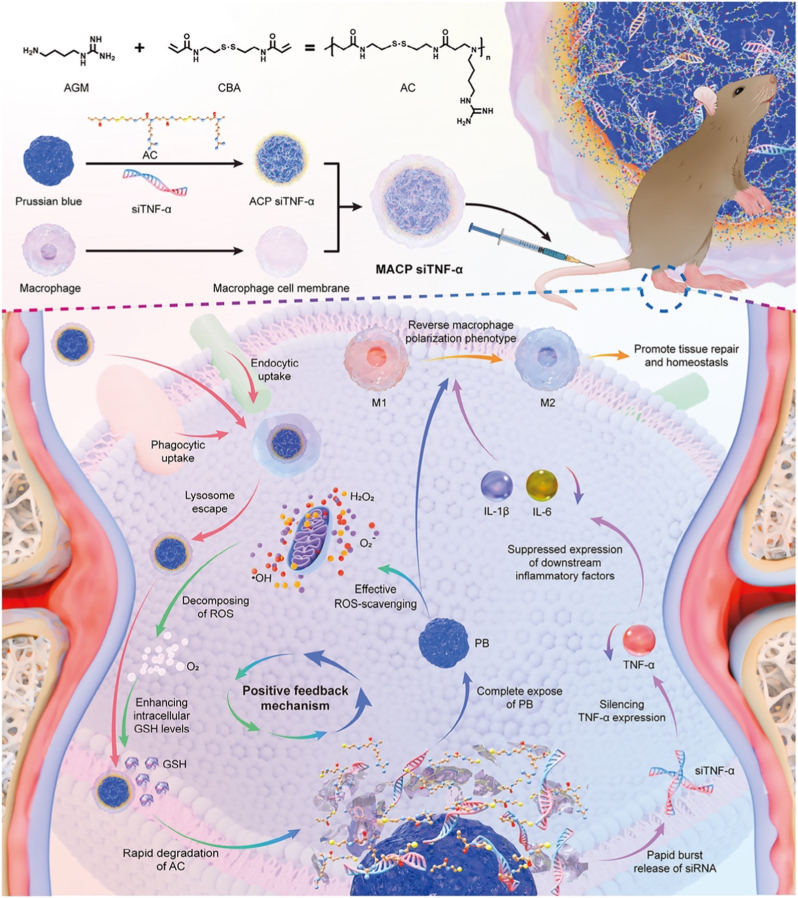
Mechanism of the biomimetic nanodelivery platform (MACP siTNF-*α* NPs) for RA treatment. Reproduced with permission from Ref. [42]. Copyright 2025 Wiley-VCH GmbH.

Macrophage-driven inflammation plays a central role in the progressive joint destruction and systemic complications characteristic of the disease. The infiltration of pro-inflammatory macrophages into the synovial niche leads to the release of a range of inflammatory cytokines, including TNF-α, which promotes synovial hyperplasia, cartilage degradation, and bone erosion [149]. Traditional treatments often fail to address the underlying immune dysfunction and inflammation-driven joint damage in a targeted manner. Therefore, there is a growing interest in biomimetic nanomedicines that can modulate the immune microenvironment and offer precise therapeutic intervention. Specifically, macrophage MCNPs have gained significant attention for their ability to target inflamed tissues and their immune-evasive characteristics, making them a promising approach to treating chronic inflammatory conditions like RA [150]. For example, the study by Xie et al. developed a biomimetic nanoplatform, MACP siTNF-α NPs, for targeted RA therapy (Fig. 11) [42]. The NPs were constructed by sequentially modifying Prussian blue (PB) nanozymes and siRNA with guanidinium-based polymers (AC) and macrophage membranes [42]. This design enabled targeted delivery to inflamed joints, preferential uptake by M1 macrophages, and lysosomal escape [42]. The system operated via a self-sustaining positive feedback mechanism: PB nanozymes scavenged ROS and restored glutathione (GSH) levels, while GSH degraded AC to release siRNA, silencing TNF-α expression [42]. This dual action reversed macrophage polarization (M1 to M2), reduced pro-inflammatory cytokines, and alleviated joint damage in a CIA model, demonstrating potent therapeutic efficacy with minimal toxicity [42]. The biomimetic approach enhanced targeting and biocompatibility, offering a promising strategy for RA treatment.

Beyond immunomodulation, gene silencing using RNA-based therapeutics represents a potent approach for RA intervention, and macrophage MCNPs offer a robust delivery vehicle to enhance targeting and minimize off-target effects. Yang et al. developed a biomimetic, carrier-free nanoplatform (M-EHPS) for targeted RA therapy. The NPs were formed by co-assembling epigallocatechin gallate (EGCG), histidine (His), and phenylboronic acid pinacol ester (PBAP) with TNF-α siRNA via hydrogen bonding, electrostatic interactions, and hydrophobic forces, eliminating the need for cationic carriers. The NPs were further coated with M1 macrophage membranes (M1M) to enhance immune evasion and inflammatory targeting. M-EHPS responded to high ROS levels in RA joints, where PBAP degradation disrupted hydrophobic interactions, triggering siRNA release. Simultaneously, EGCG scavenged reactive oxygen/nitrogen species (RONS), while siRNA silenced TNF-α, synergistically reprogramming M1 macrophages to an anti-inflammatory M2 phenotype. In RA mice, M-EHPS accumulated in inflamed joints, reduced pro-inflammatory cytokines (TNF-α, IL-1β, IL-6), and alleviated joint damage with minimal toxicity, demonstrating a dual therapeutic strategy combining gene silencing and antioxidant effects.

### 3.6 Tetrahedral framework nucleic acids

tFNAs represent a novel class of DNA nanostructures that offer a highly programmable and biocompatible platform for gene delivery. Due to their well-defined geometry, structural stability, and ease of functionalization, tFNAs exhibit excellent cellular uptake and low immunogenicity, making them particularly suited for treating inflammatory joint diseases such as OA and RA [151,152]. tFNAs can be loaded with genetic materials such as siRNA or miRNA through covalent conjugation or physical encapsulation, ensuring efficient intracellular delivery while protecting the cargo from enzymatic degradation. Once internalized by target cells, such as FLSs or chondrocytes, tFNAs facilitate endosomal escape and enable sustained gene silencing or modulation of gene expression [107,153,154].

One of the key advantages of tFNAs lies in their ability to physically encapsulate or hybridize genetic cargo without the need for chemical modification. This is primarily achieved through Watson-Crick base pairing, where complementary sequences on the nucleic acid strands enable the stable and reversible binding of small RNAs such as siRNA or miRNA onto the TFNA scaffold. This non-covalent approach not only preserves the bioactivity of the therapeutic nucleic acids but also enhances their stability, cellular uptake, and resistance to enzymatic degradation [155]. Building upon this principle, Liao et al. developed a tFNA-based siRNA delivery system, termed Tsi, for treating TMJOA [105]. The Tsi was synthesized by self-assembling four single-stranded DNAs into tFNA, followed by loading siRNA targeting NF-κB via complementary base pairing [105]. This nanostructure exhibited enhanced stability, cellular uptake, and prolonged joint retention compared to free siRNA [105]. Mechanistically, Tsi silenced NF-κB, attenuating inflammation and oxidative stress while activating the NRF2/HO-1 pathway [105]. *In vivo*, Tsi significantly reduced cartilage degradation, suppressed apoptosis, and promoted matrix regeneration in TMJOA rats [105]. The study highlights tFNA as an efficient nanocarrier for siRNA delivery, offering a promising therapeutic strategy for TMJOA.

In addition to physical hybridization, covalent conjugation offers an alternative and robust strategy for loading genetic cargo onto tFNAs. This method typically involves the chemical linkage of nucleic acids (e.g., miRNAs or siRNAs) to one of the DNA strands at a predefined site, such as the 3′ or 5′ terminus. Covalent attachment ensures precise control over loading stoichiometry, enhances structural stability, and prevents premature release of the therapeutic payload. Leveraging this approach, Wang et al. developed a tFNA-based miRNA delivery system, termed T-23b, for treating RA [106]. The T-23b complex was synthesized by conjugating miR-23b to the 3′ end of one tFNA strand via self-assembly of four single-stranded DNAs, forming a stable tetrahedral nanostructure (∼12.9 nm) [106]. The tFNA carrier enhanced miR-23b stability against enzymatic degradation and prolonged its retention in FLSs, enabling efficient cellular uptake [106]. T-23b suppressed synovial inflammation by downregulating TNF-α, IL-1β, and MMP13, inhibited osteoclast activity, and preserved cartilage matrix in RA mice [106]. Compared to free miR-23b or tFNAs alone, T-23b demonstrated superior therapeutic efficacy, significantly reducing joint swelling and systemic inflammation [106]. This study highlights tFNAs as a versatile platform for miRNA delivery in RA treatment.

In summary, tFNAs have emerged as a versatile and biocompatible platform for gene delivery, offering unique structural and functional advantages. Their well-defined geometry, nanoscale size, and intrinsic cellular uptake capacity enable efficient penetration into target cells without the need for transfection agents. tFNAs can load nucleic acids either through physical adsorption, via complementary base pairing, or chemical conjugation, ensuring enhanced stability and protection against enzymatic degradation. These features collectively improve the retention, bioavailability, and therapeutic performance of siRNA or miRNA cargos. As demonstrated in recent studies, tFNA-based systems can effectively modulate inflammatory signaling, reduce joint damage, and promote tissue regeneration, highlighting their great potential in treating inflammatory diseases such as RA and OA.

## 4 Emerging therapeutic strategies in arthritis gene therapy

Arthritis is characterized by chronic inflammation, immune dysregulation, and progressive joint destruction. Gene therapy has emerged as a transformative approach to address these multifaceted pathologies by targeting specific molecular and cellular mechanisms. Current strategies focus on reshaping the immune microenvironment (e.g., enhancing Treg activity), reprogramming synovial macrophage function, and inhibiting pro-inflammatory responses (e.g., silencing IL-1β, TNF-α). Additionally, interventions aim to suppress pathological synovial activation, protect cartilage matrix from degradation (e.g., targeting MMPs or ADAMTS-5), and promote cartilage repair (e.g., via SOX9 or TGF-β delivery). Beyond structural repair, gene therapy also addresses pain modulation by targeting neuronal sensitization pathways. This section will explore these targeted strategies, highlighting recent breakthroughs and translational challenges in the field.

### 4.1 Immune modulation

Immune dysregulation lies at the core of autoimmune arthritis pathogenesis, where the breakdown of self-tolerance leads to chronic inflammation and progressive joint damage [6]. A central therapeutic goal in gene-based approaches is the re-establishment of immune homeostasis by restoring tolerance toward self-antigens. Treg, as key mediators of peripheral tolerance, play a crucial role in suppressing autoreactive immune responses. Enhancing Treg function or expanding their population can dampen pathogenic T and B cell activity, thereby reducing tissue-destructive inflammation [156]. Concurrently, the modulation of B cell responses has emerged as an equally important axis of intervention. Strategies targeting B cell hyperactivation involve the upregulation of inhibitory receptors such as FcγRIIB, which negatively regulate B cell signaling, and the reduction of autoreactive B cells and long-lived plasma cells that sustain autoantibody production [157]. Furthermore, tol-APCs can be induced *in vivo* by delivering immunoregulatory signals, such as PDL1-encoding mRNA, which promotes T cell anergy and antigen-specific tolerance [79,158]. Collectively, these immune-modulating strategies offer a multipronged approach to recalibrate immune responses, suppress autoimmunity, and create a more tolerogenic microenvironment in rheumatoid and related forms of inflammatory arthritis. Nanotechnology enables the precise delivery of these gene modulators to immune organs and cell populations, thereby enhancing therapeutic efficacy while minimizing systemic immunosuppression.

Within the broader strategy of immune modulation, targeting dysregulated B cell activity, particularly within the spleen, a central organ for peripheral immune regulation, has gained increasing attention for the treatment of autoimmune arthritis. The spleen plays a pivotal role in maintaining B cell tolerance and modulating systemic immune responses. Achieving targeted delivery of immunomodulatory signals to the spleen enables selective reprogramming of pathogenic B cells while minimizing systemic immunosuppression [159]. In this context, Liu et al. developed spleen-targeted polymer-lipid nanoparticles (LPNs) composed of AMB-POC18 lipidoid and PEG-PLA to deliver FcγRIIB mRNA (mFcγRIIB) for RA therapy [160]. Upon intravenous (*i*.*v*.) injection, LPNs are directed toward the spleen through complement C3 adsorption, thereby selectively upregulating FcγRIIB expression in splenic B cells, suppressing hyperactivation via the FcγRIIB/Lyn/SHP-1 inhibitory pathway [160]. In a CIA model, this approach reduced autoimmune responses, decreased pro-inflammatory cytokines (TNF-α, IL-6), and ameliorated joint damage, demonstrating potent immunomodulation by restoring B cell tolerance [160]. The study highlights a precise mRNA delivery strategy targeting immune dysregulation in RA.

Building upon the concept of spleen- and B cell-directed immunomodulation, a complementary approach involves the precise depletion of pathogenic B cell subsets and long-lived plasma cells that drive autoantibody production and tissue damage in autoimmune arthritis [161]. Rather than broadly suppressing the immune system, this strategy aims to selectively eliminate autoreactive immune components while preserving protective immunity. In this vein, Guo et al. developed lipid nanoparticle-encapsulated mRNA-encoding anti-CD19 antibodies (mRNAb-LNPs) to target autoreactive B cells and plasma cells in systemic lupus erythematosus (SLE) and RA [96]. The mRNAb-LNPs were prepared by encapsulating heavy- and light-chain mRNAs of anti-CD19 IgG in ionizable lipid-based NPs (∼100 nm) [96]. Upon intramuscular injection, mRNAb-LNPs enabled sustained *in vivo* production of anti-CD19 antibodies, depleting CD19^+^ B cells and CD19^+^CD138^+^ plasma cells in MRL/lpr and CIA mouse models [96]. This targeted immunomodulation significantly reduced autoantibodies (e.g., anti-dsDNA), mitigated tissue damage (skin, kidneys, joints), and improved disease scores, demonstrating potent efficacy in resetting aberrant humoral immunity [96]. The approach leverages CD19 as a key B-cell lineage marker, offering a promising alternative to conventional therapies with enhanced pharmacokinetics and translational potential.

**Fig. 12:**
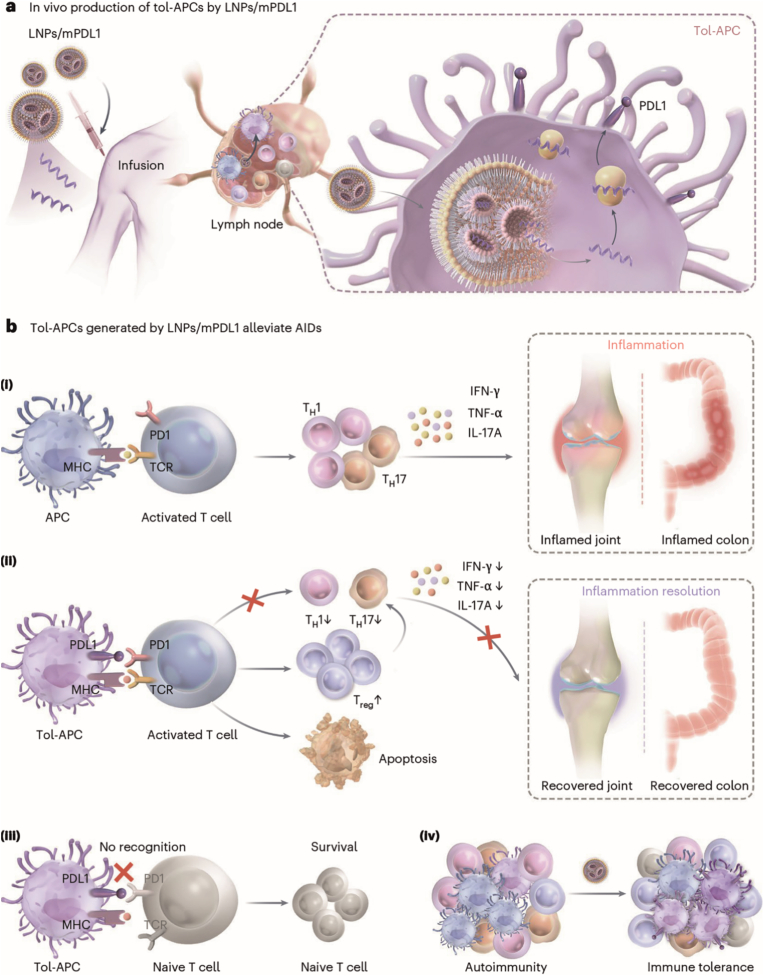
*In vivo*-produced tol-APCs by LNPs/mPDL1 selectively target activated T cells for the treatment of autoimmune diseases. Reproduced with permission from Ref. [52]. Copyright 2025 Springer Nature.

In addition to directly targeting lymphocyte subsets, an emerging immunomodulatory strategy focuses on reprogramming APCs to promote peripheral tolerance [78,80]. By enhancing the expression of inhibitory ligands on APCs, such as PDL1, it is possible to suppress autoreactive T cell activation while preserving immune homeostasis [79,158]. This strategy avoids the need for prior identification of specific autoantigens, making it broadly applicable across autoimmune conditions. Building on this concept, Liu et al. developed a low-immunogenic LNP formulation encapsulating mRNA encoding PDL1 to generate tol-APCs *in vivo* (Fig. 12) [52]. The LNPs were optimized via a design-of-experiment approach, reducing the N/P ratio and adjuvant properties to minimize co-stimulatory molecule upregulation [52]. Upon subcutaneous delivery, LNPs/mPDL1 selectively targeted APCs, inducing PDL1 expression, which suppressed activated T cells via PD1/PDL1 interaction while sparing naive T cells [52]. In mouse models of RA and UC, this approach reduced pro-inflammatory cytokines (e.g., TNF-α, IFN-γ), promoted Treg expansion, and alleviated disease progression [52], demonstrating a potent antigen-nonspecific immunomodulatory strategy for AIDs.

In summary, immune modulation represents a pivotal strategy in gene-based therapies for arthritis, aiming to restore immune tolerance and suppress pathogenic immune responses. By enhancing Treg function, reprogramming APCs toward a tolerogenic phenotype, and selectively depleting autoreactive B cells and plasma cells, these approaches address the root causes of chronic inflammation and autoimmunity. Advances in mRNA nanotechnology, particularly LNP platforms, have enabled precise delivery of immunomodulatory genes to key immune organs and cell subsets, resulting in reduced systemic inflammation, restored immune balance, and improved disease outcomes. These findings collectively underscore the therapeutic potential of nanomedicine-enabled immune modulation in the treatment of arthritis.

### 4.2 Reprogramming of macrophages

Synovial macrophages play a central role in the pathogenesis of arthritis by mediating chronic inflammation and contributing to joint destruction. These macrophages exist in two main phenotypes: the pro-inflammatory M1 macrophages and the anti-inflammatory M2 macrophages. M1 macrophages are primarily involved in the production of pro-inflammatory cytokines such as TNF-α, IL-1β, and IL-6, which drive the inflammatory cascade in AIDs like RA. In contrast, M2 macrophages exhibit immunosuppressive and tissue-repair functions, contributing to the resolution of inflammation and the promotion of tissue regeneration [162]. Therefore, reprogramming synovial macrophages to shift from a pro-inflammatory M1 phenotype to an anti-inflammatory M2 phenotype holds significant therapeutic promise. Recent strategies focus on leveraging nanotechnology to specifically modulate macrophage polarization, thereby alleviating inflammation and enhancing tissue repair in arthritic joints. By delivering targeted gene therapies or bioactive molecules to macrophages, these approaches aim to suppress M1 polarization while promoting M2 macrophage activity [42,50]. The challenge lies in designing delivery systems that can efficiently target synovial macrophages, overcome the complex immune microenvironment, and achieve precise control over macrophage polarization. These strategies not only hold potential for mitigating inflammation but also for promoting cartilage repair and joint function restoration in arthritis patients.

**Fig. 13:**
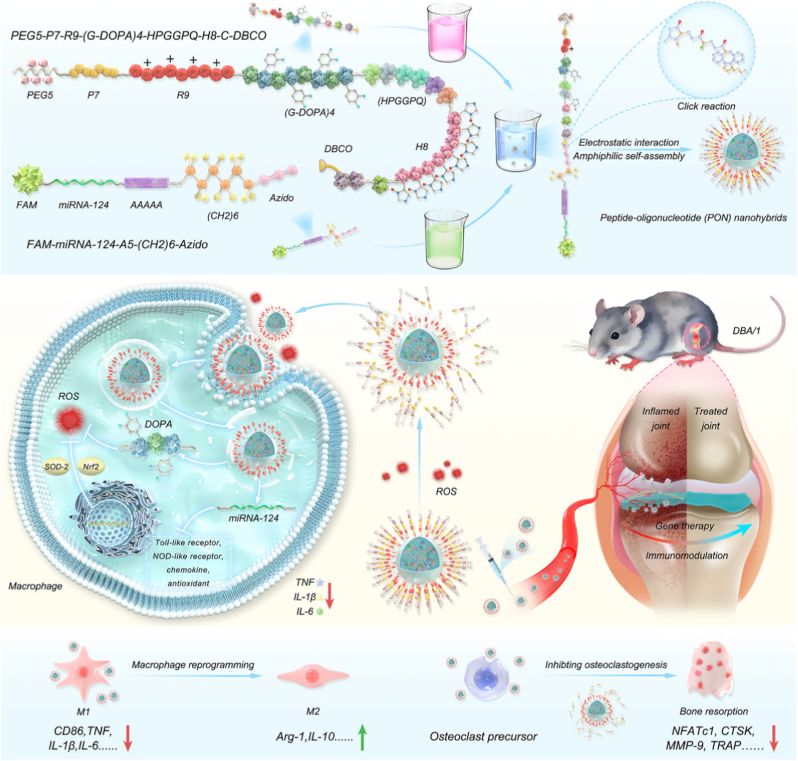
Schematic illustration showing the fabrication and function of PON nanohybrids for RA gene therapy. Reproduced with permission from Ref. [164]. Copyright 2025 Wiley-VCH GmbH.

Modulating the signaling pathways involved in inflammation and immune response is a key therapeutic strategy. Among these, the NF-κB and MAPK pathways are central regulators of macrophage polarization and the production of pro-inflammatory cytokines. In particular, these pathways are heavily involved in the activation of M1 macrophages, which contribute to the persistent inflammation and joint destruction seen in RA [163]. Therefore, targeting these signaling pathways to inhibit M1 macrophage polarization, while simultaneously promoting the M2 anti-inflammatory phenotype, can help in controlling inflammation and preventing tissue damage in the joints. Wang and colleagues developed peptide-oligonucleotide (PON) nanohybrids for targeted gene therapy of RA (Fig. 13) [164]. The nanohybrids were constructed via bioorthogonal conjugation of a ROS-cleavable peptide (PEG5-P7-R9-(G-DOPA)4-HPGCPQ-H8-C-DBCO) with anti-inflammatory miRNA-124 [164]. Upon reaching inflamed joints, ROS triggers the removal of the PEG5-P7 shield, exposing the R9 cell-penetrating peptide for selective macrophage uptake [164]. Intracellularly, cathepsin K cleaves the HPGCPQ linker, releasing miRNA-124 to silence pro-inflammatory genes (TNF-α, IL-6, iNOS) and suppress osteoclastogenic factors (NFATc1, CTSK) via inhibition of NF-κB and MAPK pathways [164]. This dual action reprograms M1 macrophages toward an M2 anti-inflammatory phenotype while blocking osteoclast differentiation [164]. In CIA mice, the nanohybrids effectively reduced joint inflammation, bone erosion, and systemic osteoporosis [164], demonstrating both therapeutic and prophylactic potential through macrophage modulation and precise gene targeting.

Building upon the strategic inhibition of pro-inflammatory pathways such as NF-κB and MAPK in RA, a similar approach can be applied to OA by modulating macrophage polarization and enhancing tissue regeneration. OA, characterized by chronic inflammation and cartilage degradation, involves the activation of inflammatory pathways, including p38 MAPK, NF-κB, and MyD88, which are central to the polarization of macrophages into the pro-inflammatory M1 phenotype [165]. Targeting these pathways can effectively reduce inflammation and prevent further cartilage damage. Cui et al. developed AHK-CaP/siCA9 NPs for OA therapy by co-delivering kartogenin (KGN) and carbonic anhydrase IX (CA9)-targeting siRNA [166]. The NPs were synthesized via nanoprecipitation of HA-alendronate-KGN (AHK) polymers with CaP-condensed siRNA, forming a core-shell structure (220 nm) [166]. The NPs selectively targeted synovial macrophages, where siCA9 silenced CA9 to repolarize pro-inflammatory M1 macrophages to anti-inflammatory M2 phenotypes by downregulating IL-6/TNF-α and upregulating IL-10/CD206 via p38 MAPK, NF-κB (p50/p65), and MyD88 signaling pathways’ inhibition [166]. Concurrently, KGN promoted chondrogenic differentiation of MSCs by upregulating TGF-β1/ACAN/COL2α1 [166]. In OA mice, the NPs synergistically attenuated synovitis, reduced cartilage degradation (lower OARSI scores), and enhanced regeneration by modulating macrophage polarization and MSC differentiation [166]. The dual-action strategy demonstrated efficacy in both early and advanced OA models [166].

Additionally, a promising therapeutic strategy involves manipulating intracellular signaling pathways, particularly those regulated by the endoplasmic reticulum (ER) stress sensor ERN1. This approach aims to shift macrophages from a pro-inflammatory M1 phenotype to a reparative M2 phenotype, which is crucial for resolving inflammation and promoting tissue repair. A key aspect of this polarization involves the modulation of intracellular calcium levels, which are regulated through the interaction of ERN1 with inositol 1,4,5-trisphosphate receptors (IP3R1/3) [167,168]. Disrupting this signaling cascade has shown significant potential for therapeutic intervention. Guo et al. developed a macrophage-targeting siERN1-nanoprodrug (FA-PEG-R-NPs@siERN1) by conjugating siRNA against ERN1 (siERN1) with a disulfide-containing cationic core (ss-PBAA-PEI), a cell-penetrating peptide (RKKRRQRRR), PEG for stability, and folic acid (FA) for macrophage specificity [169]. The nanoprodrug exhibited pH/redox-responsive release and efficiently delivered siERN1 to macrophages [169]. Mechanistically, siERN1 downregulated IRE1α (encoded by ERN1), disrupting its interaction with IP3R1/3, thereby reducing intracellular Ca^2+^ levels and promoting M2 macrophage polarization [169]. Additionally, it inhibited MyD88-dependent TLR signaling, suppressing pro-inflammatory cytokines (TNF-α, IL-6, IL-1β) [169]. In CIA and inflammatory bowel disease (IBD) models, the nanoprodrug alleviated inflammation, reduced joint/cartilage damage, and restored immune homeostasis by balancing Th17/Treg cells and M1/M2 macrophages [169].

In summary, synovial macrophage reprogramming represents a promising strategy for treating inflammatory joint diseases, such as RA and OA. By targeting specific intracellular signaling pathways, such as the NF-κB, MAPK, and MyD88 pathways, as well as manipulating ER stress responses, it is possible to shift macrophages from a pro-inflammatory M1 phenotype to a reparative M2 phenotype. This reprogramming not only reduces inflammation but also enhances tissue regeneration, offering a multifaceted approach to disease management. The use of targeted NPs or nanoprodrugs to deliver specific modulators, such as miRNAs or siRNAs, to macrophages provides an efficient means to achieve precise and sustained therapeutic effects, paving the way for advanced treatments with improved clinical outcomes.

### 4.3 Regulation of inflammatory signaling

Inflammation is a hallmark of both RA and OA, driven by the overproduction of pro-inflammatory cytokines such as IL-1β, TNF-α, and IL-6. These cytokines play a central role in synovial inflammation, cartilage destruction, and bone erosion [170,171]. Gene therapy strategies targeting these cytokines have shown significant potential in preclinical and clinical studies. Conversely, enhancing anti-inflammatory cytokines like IL-10 offers a complementary approach by promoting resolution of inflammation and tissue repair [172].

TNF-α is a pivotal pro-inflammatory cytokine deeply implicated in the pathogenesis of RA. Secreted primarily by activated macrophages, TNF-α initiates and sustains synovial inflammation through multiple mechanisms. It promotes the expression of adhesion molecules on endothelial cells, thereby enhancing leukocyte recruitment to inflamed joints [173]. TNF-α also upregulates other inflammatory mediators such as IL-1β and IL-6, amplifying the local cytokine storm. Additionally, it stimulates synoviocytes and osteoclasts, leading to pannus formation, cartilage degradation, and bone erosion, which are hallmark features of joint destruction in RA [174]. Given its central role in orchestrating inflammatory and destructive pathways, TNF-α has become a key therapeutic target. While biologics targeting TNF-α protein (e.g., infliximab, etanercept) have revolutionized RA treatment, they are associated with limitations such as immunogenicity, systemic immunosuppression, and high cost [175]. In contrast, RNA-based therapies, particularly siRNA, offer a more precise approach by downregulating TNF-α at the mRNA level [176]. This allows for selective silencing of cytokine production at the source, minimizing off-target effects and potentially allowing for safer and more sustainable inflammation control. For example, Guo and colleagues developed a novel nanomedicine for RA therapy based on a metal-organic framework (MOF) composed of tannic acid and Fe^3+^, which was used to load anti-TNF-α siRNA via sonication [177]. The NPs (TFSB) were further modified with bovine serum albumin (BSA) for targeted delivery to inflamed joints and M1-type macrophages [177]. The MOFs not only enabled high siRNA loading and protection from degradation, but also exhibited strong RONS scavenging abilities [177]. The combined effects of siRNA-mediated cytokine silencing and MOF-mediated oxidative stress reduction synergistically promote M1-to-M2 macrophage repolarization and attenuate inflammation [177]. In CIA mice, TFSB achieved effective accumulation at the disease site and significantly reduced joint inflammation and tissue damage, showing superior therapeutic efficacy and biocompatibility [177]. This multifunctional system demonstrates great potential for RA treatment by integrating gene therapy and immunomodulation.

**Fig. 14:**
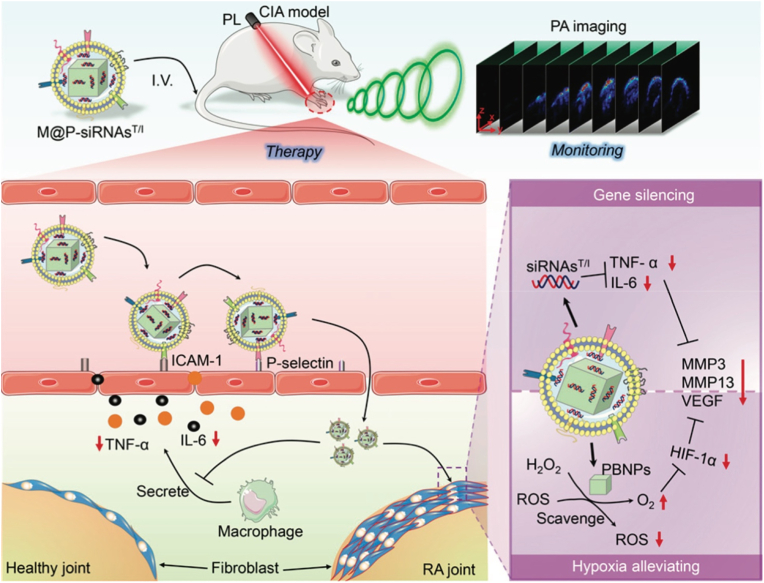
Basic schematic showing how the M@P-siRNAs^T/I^ of the present study could function as a PA imaging-guided RA therapeutic. Reproduced with permission from Ref. [182]. Copyright 2022 PNAS.

IL-6 is a multifunctional cytokine that plays a central role in the pathogenesis of RA [178]. It is abundantly produced by activated macrophages and FLSs in the inflamed joint and contributes to both local and systemic disease features. Locally, IL-6 promotes synovial hyperplasia, inflammatory cell infiltration, angiogenesis, and osteoclast differentiation, all of which contribute to joint destruction [179]. Systemically, IL-6 induces hepatic acute-phase protein production, contributing to systemic inflammation, anemia, and fatigue commonly observed in RA patients [180]. The widespread activity of IL-6 makes it a compelling therapeutic target. RNAi using siRNA offers an alternative approach by silencing IL-6 expression at the mRNA level, thus preventing downstream pathological cascades at the source. This method allows for precise, localized, and reversible modulation of cytokine levels, potentially reducing systemic immunosuppression and side effects. Importantly, IL-6 and TNF-α often act synergistically to drive RA pathogenesis. TNF-α not only directly induces joint inflammation but also stimulates IL-6 production, creating a feed-forward inflammatory loop [181]. Simultaneous suppression of both cytokines has been shown to produce superior anti-inflammatory effects compared to targeting either alone, as it disrupts multiple levels of the cytokine network and reduces the risk of compensatory upregulation. For example, Chen et al. developed a macrophage-biomimetic nanoparticle system (M@P-siRNAs^T/I^) for RA therapy (Fig. 14) [182]. The NPs were prepared by encapsulating Prussian blue nanoparticles (PBNPs) and dual-targeted siRNAs (against TNF-α and IL-6) into macrophage membrane vesicles via extrusion [182]. This design conferred targeting ability, biocompatibility, and protection of siRNAs from degradation [182]. The NPs exhibited strong ROS scavenging via PBNPs and gene silencing via siRNAs, effectively suppressing inflammation and alleviating joint hypoxia [182]. The system also enabled near-infrared photoacoustic imaging for real-time, noninvasive monitoring [182]. In CIA mouse model, M@P-siRNAs^T/I^ significantly reduced inflammatory cytokine expression, suppressed synovial angiogenesis and fibroblast invasiveness, improved oxygenation, and prevented bone erosion, outperforming conventional controls [182]. This work highlights a synergistic approach integrating imaging, immunomodulation, and RNAi for precise and effective RA therapy.

IL-10 is a potent anti-inflammatory cytokine that plays a critical role in restraining immune responses and maintaining tissue homeostasis. It is primarily secreted by Tregs, macrophages, and dendritic cells, and functions to counterbalance pro-inflammatory cytokines such as TNF-α, IL-1β, and IL-6 [172]. IL-10 inhibits antigen presentation, suppresses the expression of major histocompatibility complex (MHC) class II molecules, and reduces the production of inflammatory cytokines by macrophages and other immune cells [183]. This makes IL-10 a key mediator of immune tolerance, essential for preventing chronic inflammation and autoimmunity. Unlike therapies that suppress individual inflammatory signals, IL-10 exerts broad regulatory effects, promoting the resolution phase of inflammation and tissue repair [184]. Nucleic acid-based strategies, such as mRNA, and pDNA delivery, offer an appealing alternative by enabling localized, sustained, and controllable expression of IL-10 at the site of inflammation. For example, Watkins et al. developed a targeted gene therapy approach using pDNA encoding IL-10 to treat OA [81]. The IL-10 plasmid was formulated for intra-articular injection, enabling localized expression within inflamed joints [81]. This strategy aimed to harness IL-10's anti-inflammatory properties to counteract pro-inflammatory cytokines and reduce pain [81]. The DNA was delivered using a polymer-based carrier that ensured stability and effective uptake in synovial tissues [81]. In preclinical OA models, IL-10 gene delivery significantly reduced pain-related behaviors, synovial inflammation, and cartilage degradation, while preserving joint structure [81]. Toxicological analysis confirmed excellent biosafety, with no adverse effects on major organs or immune markers [81].

In summary, regulating inflammatory signaling through nucleic acid-based therapies offers a promising strategy for treating RA and OA. Targeting pro-inflammatory cytokines like TNF-α and IL-6 via siRNA, alongside enhancing anti-inflammatory cytokines such as IL-10 through gene delivery, has demonstrated significant therapeutic potential in preclinical models. These approaches, especially when combined with advanced nanocarrier systems, enable precise, localized, and sustained modulation of the inflammatory microenvironment.

### 4.4 Intervention of synovial pathological activation

The pathological activation of FLSs, along with the processes of angiogenesis and immune cell infiltration, plays a central role in the progression of inflammatory joint diseases such as RA. FLSs in the synovium are critical drivers of joint inflammation, as they become hyperactivated under inflammatory conditions, contributing to cartilage degradation and bone erosion [185,186]. One of the key factors involved in this pathological activation is the FAP, which is upregulated in inflamed synovium and is associated with disease severity [187,188]. Additionally, the vascular endothelial growth factor (VEGF) signaling pathway, which promotes angiogenesis, is another pivotal player in synovial tissue remodeling and inflammation [189]. These pathological processes are often further exacerbated by pro-inflammatory cytokines, such as IL-17, which enhances the production of inflammatory mediators by FLSs and promotes immune cell infiltration [186,[190], [191], [192]]. Moreover, NF-κB signaling is a major regulator of the inflammatory response in synovial tissue, controlling the expression of key molecules involved in tissue destruction and immune cell recruitment [193]. Targeting these signaling pathways to intervene in the pathological activation of synovial cells offers a promising therapeutic strategy for halting disease progression and restoring synovial homeostasis. By modulating these key molecular pathways, it is possible to reduce inflammation, angiogenesis, and immune cell infiltration, ultimately improving joint function and preventing further tissue damage in diseases like RA.

The pathological activation of FLSs and the subsequent tissue destruction in RA are tightly regulated by various signaling pathways, including the PI3K/AKT pathway, which plays a critical role in promoting cell survival, proliferation, and inflammation. FLSs, upon activation, can proliferate abnormally, invade surrounding tissues, and contribute to pannus formation, which exacerbates joint damage [194]. Additionally, the activation of this pathway enhances angiogenesis, further fueling inflammation and tissue destruction [195]. One promising therapeutic strategy to address this issue involves the modulation of these signaling pathways through the delivery of specific genetic material, such as mRNA encoding tumor suppressor proteins. For example, Chen et al. developed a genetically engineered biomimetic NP, MR@P-mPTEN, for targeted mRNA delivery to treat RA [53]. The NP was fabricated by coating TNF-α receptor (TNF-R1)-overexpressing macrophage membranes onto PLGA cores loaded with PTEN-encoding mRNA (mPTEN) [53]. This design enabled dual functionality: competitive binding of TNF-α to suppress inflammatory signaling and delivery of mPTEN to FLSs, restoring PTEN expression and inhibiting PI3K/AKT pathway activation [53]. In CIA mice, MR@P-mPTEN reduced synovitis, joint erosion, and pro-inflammatory cytokines (TNF-α, IL-1β, IL-6), while promoting autophagy and suppressing fibroblast invasion and angiogenesis [53]. The therapy demonstrated significant targeting efficiency, stability, and biocompatibility, offering a combined strategy to modulate synovial pathology and inflammatory cascades in RA.

**Fig. 15:**
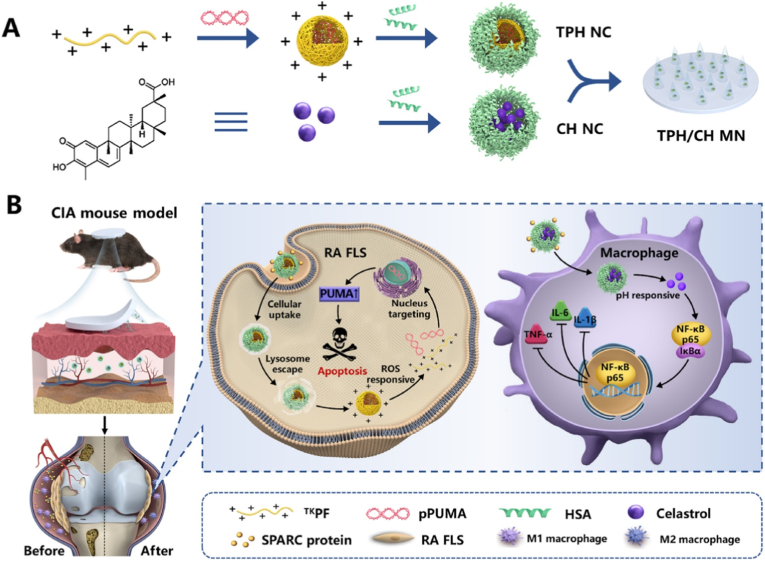
Schematic illustration of microneedle-assisted dual delivery of PUMA gene and celastrol for synergistic therapy of rheumatoid arthritis through restoring synovial homeostasis. Reproduced with permission from Ref. [198]. Copyright 2024 KeAi Communications Co. Ltd.

RA is characterized by persistent synovial inflammation, hyperplasia of FLSs, and progressive joint damage, including cartilage erosion and bone destruction. A key contributor to these pathological changes is the aberrant survival and proliferation of FLS, which invade surrounding tissues and sustain inflammatory cycles [186]. Modulating FLS apoptosis and controlling inflammation are crucial therapeutic strategies for restoring synovial homeostasis and preventing joint damage [196]. One promising approach is to induce targeted cell death in these activated FLSs by upregulating pro-apoptotic genes such as PUMA (p53 upregulated modulator of apoptosis), which can trigger apoptosis in FLSs and thereby reduce their contribution to disease progression [197]. Building on this principle, Hua et al. developed a microneedle (MN)-assisted dual-delivery system for RA therapy, combining a PUMA gene-loaded nanocomplex (TPH) and celastrol-encapsulated human serum albumin (HAS) NPs (CH) (Fig. 15) [198]. TPH was fabricated by condensing the pro-apoptotic PUMA plasmid with a ROS-responsive fluorinated polymer (TkPF) and coating it with HSA, while CH utilized HSA to encapsulate the anti-inflammatory drug celastrol [198]. The HA-based MN facilitated transdermal delivery, enabling targeted accumulation in RA joints via secreted protein acidic and rich in cysteine (SPARC)-mediated HSA affinity [198]. TPH upregulated PUMA in FLS, inducing apoptosis, while CH suppressed NF-κB activation in macrophages, reducing inflammation [198]. In a CIA model, the dual therapy synergistically restored synovial homeostasis, alleviating synovial hyperplasia, cartilage erosion, and bone destruction [198], demonstrating a potent strategy for RA treatment.

In RA, persistent inflammation and hyperplasia of the synovium, particularly in the form of FLSs and macrophages, lead to progressive joint damage, including cartilage erosion and bone destruction. The activation of key inflammatory pathways, such as NF-κB, plays a central role in sustaining these pathological processes [193,199]. NF-κB activation is tightly regulated by several factors, including TAB2, TAB3, and IKKα, which serve as upstream modulators in the NF-κB signaling cascade [200]. Inhibition of NF-κB activation can effectively suppress the production of pro-inflammatory cytokines like TNF-α, IL-6, and IL-1β, while also inducing apoptosis in hyperplastic synovial cells, providing a therapeutic approach to halt joint damage and inflammation [63]. A promising strategy to modulate this pathway involves the delivery of miRNAs that target key regulators of NF-κB signaling. One such miRNA is miR-23b, which has been shown to inhibit the activation of NF-κB through targeting TAB2, TAB3, and IKKα [201]. Han et al. developed FP-based NPs for delivering miR-23b to treat RA [98]. The FP/miR-23b NPs were synthesized by conjugating heptafluorobutyric anhydride to PAMAM dendrimers, forming stable complexes (∼230 nm) at an N/P ratio of 2.0 [98]. These NPs preferentially accumulated in inflamed joints via the extravasation through leaky vasculature and subsequent inflammatory cell-mediated sequestration (ELVIS) effect and were internalized by synovial macrophages and fibroblasts [98]. By restoring miR-23b expression, they suppressed NF-κB activation via targeting TAB2, TAB3, and IKKα, thereby inhibiting pro-inflammatory cytokine production (TNF-α, IL-6, IL-1β) and inducing apoptosis in hyperplastic synovium [98]. In RA rat and mouse models, FP/miR-23b significantly reduced synovitis, bone erosion, and cartilage damage while restoring joint function [98], demonstrating dual anti-inflammatory and pro-apoptotic efficacy without systemic toxicity.

In summary, intervening in the pathological activation of synovial tissues, particularly by targeting FLSs, macrophages, and associated inflammatory pathways, represents a promising strategy for the treatment of RA. Key mechanisms, including the inhibition of inflammatory signaling pathways such as NF-κB, VEGF, and IL-17, play pivotal roles in promoting synovial hyperplasia, joint inflammation, and tissue destruction. By targeting these pathways, therapeutic approaches aim to reduce synovial cell proliferation, suppress angiogenesis, and alleviate inflammatory responses, ultimately restoring synovial homeostasis. Recent advancements, such as the use of biomimetic NPs for targeted delivery of mRNA or genes, as well as MN-assisted dual-delivery systems, have demonstrated the potential to modulate these pathological processes effectively. Strategies that combine apoptosis induction in FLSs with the inhibition of key inflammatory pathways offer a multifaceted approach to halt the progression of RA, suggesting that such interventions may hold significant clinical promise in mitigating joint damage and improving long-term outcomes for RA patients.

### 4.5 Protection of cartilaginous matrix

OA is characterized by the progressive degeneration of articular cartilage, a process primarily driven by the imbalance between ECM degradation and repair [202,203]. The ECM, composed of collagen fibers, proteoglycans, and glycoproteins, plays a crucial role in maintaining cartilage integrity and joint function [27]. In OA, the activity of MMPs such as MMP13 and aggrecanases like ADAMTS-5 is significantly upregulated, leading to the breakdown of key ECM components, including collagen and proteoglycans. This degradation contributes to cartilage wear, joint stiffness, and pain [204,205]. Conversely, tissue inhibitors of metalloproteinases (TIMPs) serve as endogenous regulators, inhibiting the activity of MMPs and aggrecanases to maintain ECM homeostasis. However, in OA, the expression of TIMPs is often insufficient to counteract the heightened ECM degradation [206]. Given the central role of ECM degradation in OA pathogenesis, strategies that target the inhibition of MMPs, ADAMTS-5, and the restoration of TIMP balance have emerged as promising therapeutic approaches. By suppressing these catabolic enzymes or promoting the activity of TIMPs, it is possible to protect cartilage from further damage, preserve joint structure, and potentially restore normal function. Advances in nanotechnology, including the use of NPs for the targeted delivery of siRNA or miRNA to the cartilage, offer novel ways to modulate these key enzymes and enhance ECM protection. These approaches aim to limit the degradation of cartilage and slow the progression of OA, thus improving long-term joint health and function.

**Fig. 16:**
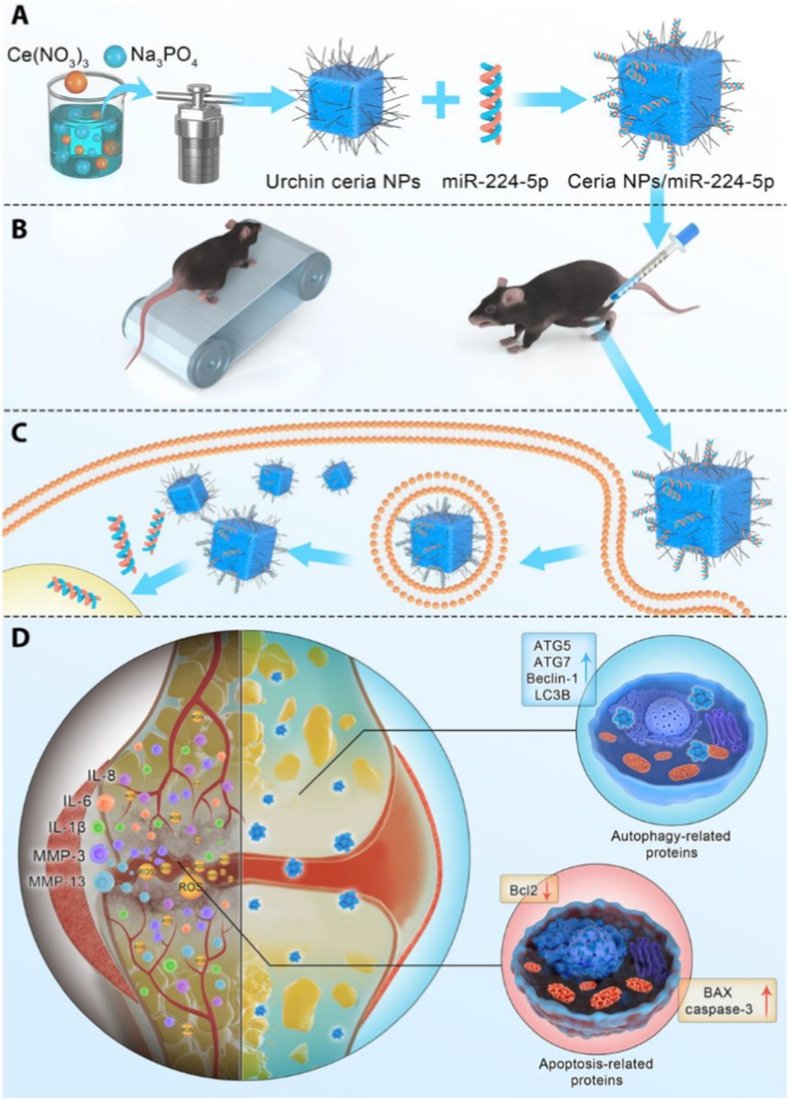
Urchin-like ceria NPs for enhanced gene therapy of OA. Reproduced with permission from Ref. [100]. Copyright 2023 American Association for the Advancement of Science.

The progressive breakdown of the ECM in OA is largely driven by the overactivation of enzymes such as MMP13 and ADAMTS, which degrade key components like collagen and proteoglycans. This degradation leads to the loss of cartilage integrity and joint function [204,205]. One promising strategy to mitigate ECM destruction involves regulating key molecular players, such as miRNAs, which can modulate the expression of enzymes involved in matrix degradation. Specifically, microRNA-224-5p (miR-224-5p) has been shown to target pentraxin 3 (PTX3), a molecule involved in ECM regulation, leading to the upregulation of CII (COL2) and aggrecan (ACAN) while inhibiting MMP13 and ADAMTS expression. This dual action can protect cartilage ECM and alleviate OA symptoms [46]. Chen et al. developed urchin-like ceria NPs loaded with microRNA-224-5p (miR-224-5p) for enhanced OA gene therapy (Fig. 16) [100]. The NPs were synthesized via hydrothermal reaction between Ce(NO_3_)_3_·6H_2_O, Na_3_PO_4_, and water, exhibiting a high Ce^4+^/Ce^3+^ ratio for ROS scavenging [100]. The urchin-like structure facilitated efficient miR-224-5p delivery into chondrocytes, targeting PTX3 to inhibit cartilage degradation [100]. The NPs protected cartilage ECM by upregulating CII and aggrecan (ACAN) while suppressing MMP13 and ADAMTs [100]. *In vivo*, the NPs alleviated OA symptoms, including synovial hypertrophy and subchondral bone sclerosis, demonstrating synergistic therapeutic effects via ROS scavenging and miR-224-5p-mediated gene regulation [100]. This approach offers a promising strategy for OA treatment by combining nanomaterial-enhanced gene delivery and microenvironment modulation.

Direct silencing of matrix-degrading enzymes such as MMP13 offers another targeted strategy to protect cartilage integrity in OA. Unlike indirect regulators such as miRNAs, siRNAs can specifically downregulate the expression of MMP13 at the mRNA level [207]. To enhance the specificity and efficacy of siRNA delivery, Bedingfield et al. developed a monoclonal antibody-functionalized nanoparticle (mAbCII-siNP) targeting CII in damaged cartilage to deliver siRNA against MMP13 [34]. The NPs were synthesized via RAFT polymerization, conjugating an anti-CII antibody to a PEGylated diblock copolymer for enhanced cartilage retention [34]. By anchoring to exposed CII in osteoarthritic joints, the mAbCII-siNPs achieved sustained MMP13 gene silencing (>90 % knockdown), reducing cartilage degradation and preserving joint integrity in PTOA mouse models [34]. The treatment suppressed MMP13-driven ECM breakdown, inflammatory gene clusters, and secondary pathologies like osteophyte formation, outperforming clinical steroids [34]. This approach highlights the potential of matrix-targeted RNAi nanomedicine to disrupt the degenerative cycle of OA by protecting cartilage and modulating disease-related gene expression.

Beyond enzymatic degradation, cellular senescence is increasingly recognized as a key contributor to cartilage matrix breakdown in OA. Senescent chondrocytes secrete pro-inflammatory cytokines and matrix-degrading enzymes as part of the SASP, further activating pathways like NF-κB and accelerating ECM destruction [208]. Targeting senescence-related signaling cascades thus represents a novel avenue for OA intervention. In this context, Zhao et al. developed a LNP delivery system loaded with siRNA targeting FAP to treat OA [95]. The LNPs were synthesized via microfluidic technology, combining ionizable lipids, cholesterol, PEG-modified polymers, and a nuclear localization peptide (NLS-SM102) to encapsulate FAP siRNA [95]. This formulation achieved efficient chondrocyte targeting and sustained siRNA release [95]. The LNP@FAP siRNA effectively silenced FAP expression in chondrocytes, reducing SASP and inhibiting NF-κB signaling, thereby protecting cartilage matrix integrity [95]. In rat OA models, intra-articular injections of LNP@FAP siRNA significantly attenuated cartilage degeneration, decreased subchondral bone loss, and suppressed synovial inflammation [95], demonstrating its potential as a disease-modifying therapy for OA by targeting FAP-positive chondrocytes.

In summary, strategies for cartilage matrix protection in OA focus on inhibiting the degradation of the ECM, which is crucial for maintaining cartilage integrity and joint function. Key targets in this approach include MMP13, aggrecanases such as ADAMTS-5, and TIMPs, which regulate ECM remodeling. Recent advancements have explored innovative gene therapy techniques, such as siRNA and miRNA delivery, to specifically target these degradation factors. Nanoparticle-based systems have been particularly effective in delivering these therapeutic agents to the affected cartilage, enhancing their stability, targeting efficiency, and sustained release. By targeting key genes like MMP13 and FAP, these therapies mitigate cartilage degradation, reduce inflammation, and preserve joint structure, offering promising disease-modifying treatments for OA. Through these strategies, there is a significant potential not only for halting cartilage breakdown but also for promoting cartilage repair and restoring joint homeostasis.

### 4.6 Promotion of cartilage regeneration

In OA, the degeneration of articular cartilage is accompanied by a limited intrinsic capacity for tissue repair, primarily due to the avascular, a neural, and low cellularity nature of cartilage. This poor regenerative potential poses a major challenge for restoring joint function in OA patients [209]. Therefore, promoting cartilage regeneration has emerged as a vital therapeutic goal, aiming to stimulate chondrogenesis, enhance ECM synthesis, and reconstruct the structural and functional integrity of cartilage. Key molecular regulators are central to driving cartilage regeneration. SOX9, a master transcription factor for chondrogenesis, plays a pivotal role in directing the differentiation of MSCs into chondrocytes and activating the expression of cartilage-specific genes such as COL2A1 and ACAN [210]. TGF-β and BMPs, particularly BMP2 and BMP7, further contribute to this process by activating Smad-dependent signaling cascades that promote chondrocyte proliferation, matrix production, and the maintenance of a healthy cartilage phenotype [211,212]. These factors collectively orchestrate the complex events required for cartilage formation, remodeling, and long-term maintenance. However, in the inflammatory and catabolic microenvironment of OA, the expression and activity of SOX9, TGF-β, and BMPs are often dysregulated, leading to impaired chondrocyte function and compromised matrix synthesis [213,214]. Therefore, strategies aimed at restoring or enhancing the activity of these regenerative factors are of great interest. Recent advances in nanotechnology and gene modulation offer powerful tools for the targeted delivery of transcription factors, growth factors, or gene-editing elements to articular cartilage, thereby reactivating regenerative pathways in situ. By precisely upregulating SOX9, TGF-β, and BMP signaling, these approaches hold the potential to overcome the intrinsic limitations of cartilage repair in OA and support functional tissue regeneration.

**Fig. 17:**
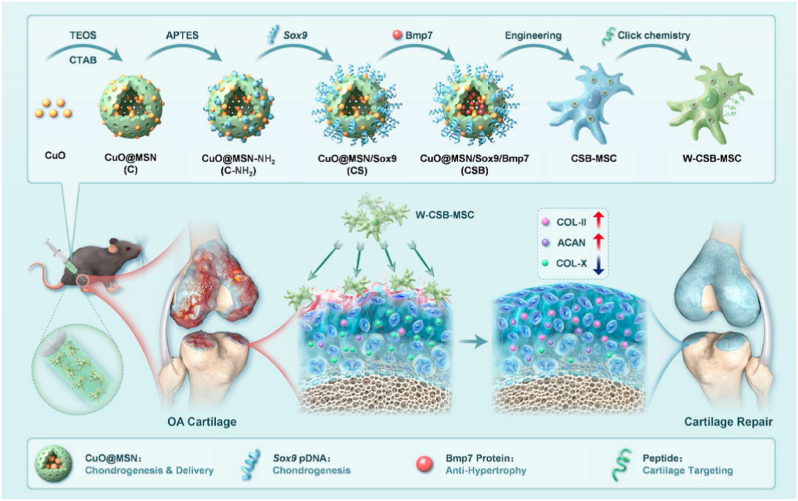
Schematic illustration for the preparation of co-delivery NPs and W-CSB-MSCs and their application in OA therapy. Reproduced with permission from Ref. [217]. Copyright 2024 Elsevier Ltd.

To effectively restore the cartilage matrix in OA, it is critical to reinitiate chondrogenic lineage commitment while simultaneously restraining aberrant hypertrophic differentiation, which often limits the regenerative potential of MSCs [215]. Central to this process is the SOX9 transcriptional axis, a master regulator of chondrogenesis that orchestrates the expression of essential ECM proteins such as CII and aggrecan (ACAN) [210]. However, in the OA milieu, the balance between chondrogenesis and hypertrophy is frequently disrupted, with elevated levels of hypertrophic markers like MMP13 and COL10A1 driving pathological cartilage remodeling [216]. Addressing this dual challenge requires a synergistic strategy that enhances SOX9-driven chondrogenic programming while concurrently suppressing pro-hypertrophic cues. In response to this therapeutic need, Wu et al. developed a dual-functional nanoparticle system, CuO@MSN/SOX9/BMP7 (CSB NPs), to enhance MSC-based cartilage repair in OA (Fig. 17) [217]. The NPs were synthesized by loading SOX9 pDNA (promoting chondrogenesis) and BMP7 protein (inhibiting hypertrophy) into aminated CuO@MSN NPs [217]. CSB NPs efficiently transfected MSCs, upregulating chondrogenic markers (e.g., SOX9, COL2A1, ACAN) while suppressing hypertrophic genes (COL10A1, MMP13) [217]. Additionally, MSCs were conjugated with a cartilage-targeting peptide (WYRGRLCCCC) to improve joint retention [217]. In OA mouse models and human cartilage explants, the engineered W-CSB-MSCs significantly enhanced hyaline cartilage regeneration and reduced hypertrophy, demonstrating a promising strategy for clinical OA therapy [217]. This approach addresses key challenges in MSC differentiation and homing for effective cartilage repair.

**Fig. 18:**
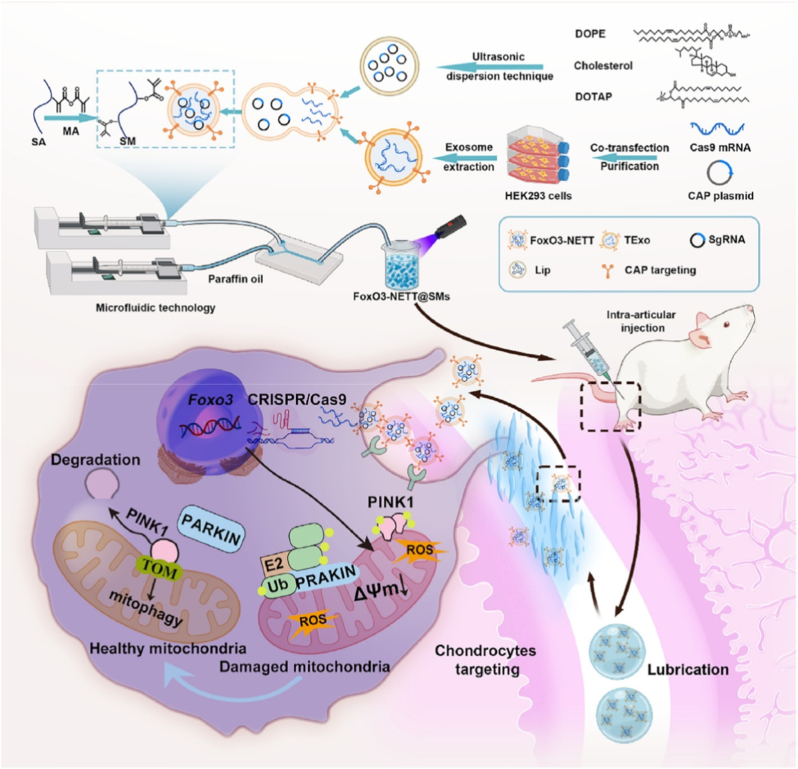
Schematic of FoxO3-NETT@SMs targeting-regulated FoxO3 gene *in vivo* to modulate mitochondrial dynamics for osteoarthritis therapy. Reproduced with permission from Ref. [222]. Copyright 2025 Chinese Pharmaceutical Association and Institute of Materia Medica, Chinese Academy of Medical Sciences.

Beyond ECM synthesis and phenotypic stabilization, the intracellular metabolic status of chondrocytes, particularly mitochondrial quality control, plays a pivotal role in OA pathogenesis. Recent evidence suggests that mitochondrial dysfunction and impaired mitophagy contribute to oxidative stress, apoptosis, and inflammation within the cartilage microenvironment, exacerbating chondrocyte degeneration and ECM breakdown [[218], [219], [220]]. A key regulatory node in this process is the transcription factor FoxO3, which governs autophagic and mitophagic flux through the PINK1/Parkin signaling axis, thereby preserving mitochondrial integrity and supporting chondrocyte survival [221]. However, targeted and sustained activation of FoxO3 in diseased joints remains a technical hurdle. To address this, Chen et al. developed a nanoengineered cargo, FoxO3-NETT@SMs, for OA therapy by encapsulating a chondrocyte-targeted CRISPR-Cas9-based FoxO3 gene-editing tool (FoxO3-NETT) within injectable sodium alginate hydrogel microspheres (MSs) (Fig. 18) [222]. The FoxO3-NETT was fabricated via fusion of FoxO3-sgRNA-loaded liposomes and Cas9 protein-enriched exosomes, enabling precise gene delivery [222]. The system upregulated FoxO3, a key regulator of mitophagy, activating the PINK1/Parkin pathway to restore mitochondrial function in chondrocytes [222]. This promoted ECM synthesis (e.g., COL2, SOX9) while suppressing inflammation and apoptosis [222]. *In vivo*, FoxO3-NETT@SMs alleviated OA progression by enhancing cartilage regeneration, reducing joint degradation, and improving gait in rat models [222], demonstrating a novel gene-targeted strategy for OA treatment.

In addition to mitochondrial homeostasis and phenotype stabilization, cellular senescence of chondrocytes and the limited recruitment of reparative progenitor cells are critical barriers to effective cartilage regeneration in OA [223]. Senescent chondrocytes exhibit increased expression of catabolic enzymes (e.g., MMP13, ADAMTS5) and cell cycle inhibitors (e.g., p21), which accelerate ECM degradation and perpetuate joint inflammation [224]. Recent studies have identified microRNA-29b-5p (miR-29b-5p) as a potent regulator of chondrocyte senescence through its inhibition of TET1, an epigenetic modulator linked to senescence-associated gene expression [225]. Moreover, enhancing the homing and differentiation of endogenous synovial mesenchymal stem cells (SMSCs) offers a promising complementary approach to replenish the chondrocyte pool and support matrix repair [226,227]. Building upon this, Zhu et al. developed an injectable stem cell-homing hydrogel (SKP@miR) for OA therapy by encapsulating miR-29b-5p agomir within a self-assembling peptide nanofiber hydrogel functionalized with the homing peptide SKPPGTSS [225]. The hydrogel was prepared by electrostatic integration of the cationic miR-29b-5p mimic with the peptide matrix, enabling sustained release and deep cartilage penetration [225]. The delivered miR-29b-5p suppressed chondrocyte senescence by downregulating TET1, reducing catabolic enzymes (MMP13, ADAMTS5) and senescence markers (P16INK4a/P21), while upregulating ECM components (COL2, aggrecan) [225]. Concurrently, the SKPPGTSS peptide recruited endogenous SMSCs, promoting their chondrogenic differentiation [225]. In a rat OA model, SKP@miR restored cartilage integrity, reduced synovitis, and improved joint function [225], demonstrating a synergistic gene/stem cell therapy for OA.

While targeting senescence-associated pathways and enhancing endogenous stem cell recruitment are vital strategies for OA treatment, precise and sustained intra-articular delivery of therapeutic genes remains a central challenge. Among various genetic regulators, microRNA-140 (miR-140) has emerged as a cartilage-specific miRNA that plays a pivotal role in maintaining cartilage homeostasis by directly inhibiting matrix-degrading enzymes, such as MMP13 and ADAMTS5 [228]. However, the therapeutic application of miR-140 is hampered by its instability in the joint environment and inefficient cellular uptake. To address these limitations, advanced delivery systems that integrate targeted gene therapy with responsive biomaterial platforms are being developed to optimize spatial and temporal control of therapeutic action [229]. In this context, Li et al. developed injectable “nano-micron” gene-hydrogel MSs for OA treatment by combining arginine/histidine/phenylalanine-modified PAMAM (G5-AHP) with miR-140, a cartilage-specific miRNA targeting MMP13 and ADAMTS5 [230]. The G5-AHP/miR-140 NPs were encapsulated in gelatin methacryloyl (GelMA) MSs fabricated via microfluidic technology, ensuring uniform size (∼150 μm) and MMP-responsive release [230]. The NPs enhanced chondrocyte uptake, protected miR-140 from degradation, and upregulated CII while suppressing catabolic enzymes (MMP13/ADAMTS5) [230]. *In vivo*, intra-articular injection in OA mice reduced cartilage degradation, osteophyte formation, and joint space narrowing, demonstrating sustained gene delivery and ECM preservation [230]. This system synergized gene therapy with biomaterial-guided release to promote cartilage repair.

In summary, recent advancements in promoting cartilage regeneration in OA focus on combining gene delivery and stem cell-based therapies. Strategies such as miRNA modulation and stem cell homing are used to enhance cartilage ECM production, suppress catabolic enzymes, and recruit endogenous stem cells. The use of hydrogel-based NPs enables sustained and targeted delivery of therapeutic agents, creating an optimal environment for cartilage repair. These approaches highlight the potential for synergistic treatments that improve cartilage regeneration and provide new avenues for effective OA therapy.

### 4.7 Modulation of arthritis-related pain

Pain relief is a critical aspect of OA management, as chronic pain significantly affects the quality of life and limits mobility. OA pain is often a result of both inflammatory processes and neurogenic changes within the joint, where nociceptive signaling and neuroplasticity contribute to the persistent pain [231]. The interaction between cartilage degradation and nerve endings in the joint can amplify pain sensations, with neuroinflammation and pain signaling molecules like nerve growth factor (NGF) and substance P (SP) playing key roles in the disease progression [232,233]. Current pain management strategies, such as anti-inflammatory drugs and opioids, have limited long-term effectiveness and are associated with significant side effects [234]. As a result, there is increasing interest in developing more targeted and sustainable pain relief strategies for OA. Recent advances focus on utilizing RNAi technologies and exosome-based delivery systems to specifically target pain-related pathways. These approaches aim to block key molecules involved in pain signaling, such as NGF or SP, and inhibit their downstream effects on pain perception. Additionally, the modulation of neuro-cartilage interactions through gene therapies, such as miRNA-based treatments, is emerging as a promising avenue to reduce pain and improve joint function in OA patients. By targeting the molecular and cellular mechanisms of pain, these novel strategies provide a potential pathway for safer and more effective long-term pain relief in OA.

**Fig. 19:**
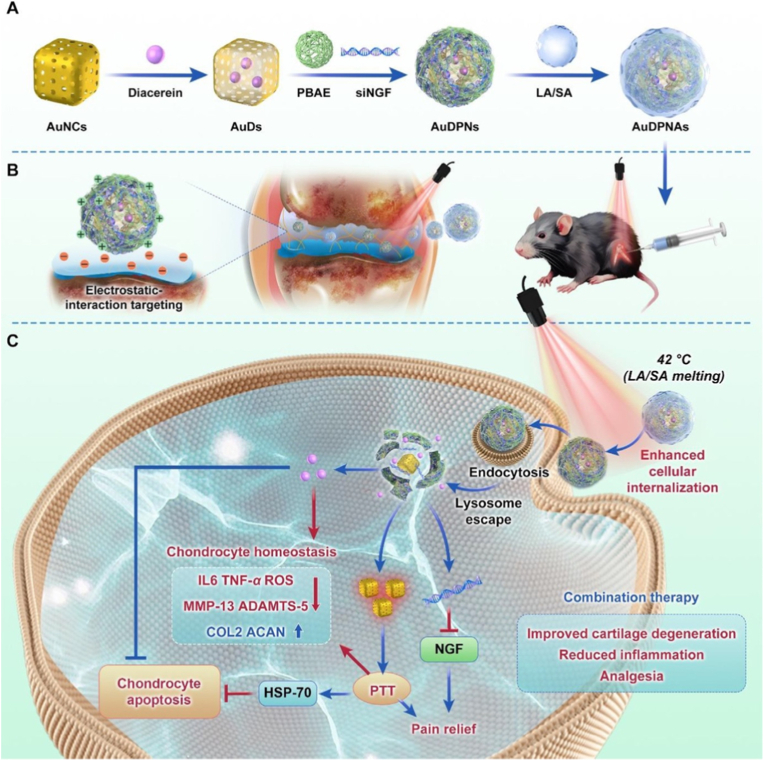
Schematic illustration of the preparation of AuNCs-based nanodrug system (AuDPNAs) for the co-delivery of DIA and siRNA, and its therapeutic mechanism for OA *in vitro* and *in vivo*. Reproduced with permission from Ref. [235]. Copyright 2024 Chinese Pharmaceutical Association and Institute of Materia Medica, Chinese Academy of Medical Sciences.

One promising approach to addressing OA-related pain involves combining anti-inflammatory therapies with RNAi strategies, targeting specific pain-related pathways to reduce pain and inflammation. Recent studies have emphasized the importance of NGF in OA pain, as it is a key mediator of neurogenic inflammation and nociceptive signaling in joint tissues [232]. To this end, Qiao et al. developed a light-inducible hybrid nanomedicine (AuDPNAs) for OA treatment, combining anti-inflammatory therapy and RNAi (Fig. 19) [235]. The nanodrug was constructed using poly(β-amino-ester)-coated gold nanocages (AuNCs) loaded with diacerein (DIA) and siRNA targeting NGF [235]. Phase-change materials (LA/SA) enabled photothermal-triggered drug release [235]. Upon intra-articular injection, AuDPNAs accumulated in joint lesions, where near-infrared light induced localized heat to promote DIA/siRNA release [235]. DIA reduced inflammation and oxidative stress, while siRNA silenced NGF, alleviating pain [235]. The nanodrug demonstrated enhanced chondroprotection, reduced cartilage degradation, and significant pain relief in OA mice [235], offering a synergistic strategy for OA therapy.

Further advancements in pain management for OA have focused on targeting the complex neuro-cartilage interactions through gene therapy strategies. One such target is the SP1-LRP1 signaling pathway, which plays a critical role in nerve ingrowth and activation within joint tissues [236]. By modulating this pathway, it is possible to reduce pain while also addressing the underlying structural damage in OA. In this regard, Lu et al. developed an exosome-mimetic (EM) nanocarrier loaded with miR-204 (EMs^miR−204^) to treat OA pain [236]. The EMs were prepared by extruding MSCs into nano-sized vesicles, followed by electroporation to encapsulate miR-204 [236]. Intra-articular delivery of EMs^miR−204^effectively targeted chondrocytes and inhibited the SP1-LRP1 signaling pathway, a key regulator of neuro-cartilage interaction [236]. By downregulating LRP1, miR-204 reduced nociceptive neuron activation and nerve ingrowth into joint tissues, alleviating mechanical allodynia and improving mobility in OA mice [236]. Additionally, EMs^miR−204^attenuated cartilage degradation by suppressing MMP13 and ADAMTS5 [236]. This study highlights miR-204 as a dual-acting therapeutic agent for OA pain and structural damage.

In summary, effective pain relief remains a cornerstone of OA treatment, as chronic pain significantly impacts the quality of life and joint function. Traditional pain management approaches often fail to address the complex mechanisms of OA-related pain, which involves both inflammatory processes and neurogenic changes in the joint. Recent advancements in therapeutic strategies have focused on more targeted approaches, such as combining anti-inflammatory treatments with RNAi or gene therapies, to block pain-related pathways and alleviate neuro-cartilage interactions. Technologies like exosome-based delivery systems and light-inducible nanomedicines offer promising solutions by precisely targeting key molecules like NGF and modulating critical signaling pathways, such as SP1-LRP1, that drive pain and cartilage degradation. These innovative strategies not only provide effective pain relief but also offer potential for joint preservation and structural repair, representing a significant step forward in the management of OA pain.

## 5 Conclusions and prospects

Gene therapy represents a transformative paradigm for the treatment of arthritis, offering the potential to correct disease-causing molecular pathways, reprogram pathogenic immune responses, and stimulate tissue regeneration. The continuous evolution of gene modulation strategies, ranging from RNA and DNA-based interventions to precise genome editing via CRISPR-Cas9, has laid a strong foundation for developing personalized and disease-modifying therapies. However, the full realization of gene therapy's potential in clinical rheumatology hinges on the ability to overcome biological barriers, ensure cell-type specificity, and minimize off-target effects. Nanotechnology has emerged as a powerful enabler, enhancing the stability, targeting accuracy, and controlled release of therapeutic genes. Advanced nanocarriers such as lipid-based NPs, polymeric systems, inorganic frameworks, and bioinspired vesicles provide versatile platforms to meet the complex demands of intra-articular delivery and systemic tolerance. Furthermore, the integration of nanodelivery with emerging therapeutic strategies, such as immune tolerance induction, synovial microenvironment remodeling, and chondroprotection, has expanded the functional scope of gene therapy in arthritis.

Looking ahead, several critical challenges must be addressed to fully realize the clinical potential of nanotechnology-enabled gene therapy for arthritis. First, the scalable and reproducible manufacturing of complex nanoplatforms remains a major hurdle, necessitating standardized protocols to ensure batch-to-batch consistency, stability, and cost-effectiveness suitable for large-scale production. Second, comprehensive long-term safety evaluations are essential to assess potential risks, including immunogenicity, toxicity, insertional mutagenesis, and off-target genome editing effects. Given the chronic nature of arthritis and the likelihood of repeated administrations, strategies to mitigate immune rejection and unintended inflammatory responses are particularly critical. In addition, systematic post-treatment safety monitoring should be emphasized, including long-term surveillance of treated patients, sensitive biomarker assays, and advanced imaging technologies to detect early adverse events or disease recurrence. Establishing standardized follow-up protocols will be indispensable for clinical translation. Third, bridging the gap between promising preclinical results and successful human clinical trials requires not only more predictive animal models and robust biomarkers for treatment efficacy but also rigorous monitoring frameworks that integrate both efficacy and safety endpoints.

Furthermore, advancing this field will rely heavily on interdisciplinary collaboration across synthetic biology, materials science, immunology, and bioengineering to refine delivery vectors with enhanced targeting specificity, stimuli-responsive release, and improved biocompatibility. Personalized medicine approaches that incorporate patient-specific genetic and immunological profiles can further tailor gene therapies to maximize efficacy while minimizing adverse effects. In parallel, the integration of nanogene therapies with existing treatment modalities, such as disease-modifying antirheumatic drugs (DMARDs), biologics, or even non-pharmacological interventions like physical therapy, offers additional opportunities to improve patient outcomes. For instance, nanoplatforms delivering therapeutic genes in combination with conventional immunomodulatory agents may achieve synergistic disease suppression, thereby reducing the required doses of systemic drugs and minimizing their associated toxicities. Co-administration with biologics targeting TNF-α or IL-6 could also enhance therapeutic durability by simultaneously modulating upstream and downstream inflammatory pathways. Moreover, coupling nanogene approaches with physical rehabilitation strategies may help preserve joint function and accelerate recovery, representing a comprehensive, multimodal treatment paradigm. Emerging technologies such as single-cell sequencing, machine learning, and advanced imaging will play pivotal roles in understanding disease heterogeneity and guiding these combination strategies in real time, enabling more precise monitoring of both efficacy and safety.

Ultimately, with sustained innovation, rigorous validation, and close integration of clinical insights, nanotechnology-powered gene therapies hold immense promise to transform arthritis treatment by enabling precise modulation of disease pathways, durable symptom relief, and potentially disease reversal. This multidisciplinary paradigm shift paves the way toward more effective, safer, and personalized interventions that could significantly improve the quality of life for millions of arthritis patients worldwide.

## CRediT authorship contribution statement

**Shuo Wang:** Writing – review & editing, Writing – original draft, Visualization, Supervision, Conceptualization. **Yuequan Wang:** Writing – review & editing, Writing – original draft, Visualization, Validation. **Qin Chen:** Supervision, Conceptualization.

## Declaration of competing interest

The authors declare that they have no known competing financial interests or personal relationships that could have appeared to influence the work reported in this paper.

## Data Availability

No data was used for the research described in the article.
